# An ABC-B Transporter Helps Protect *Fusarium graminearum* Against Enniatin Toxicity

**DOI:** 10.3390/jof12070524

**Published:** 2026-07-17

**Authors:** Linda J. Harris, Whynn Bosnich, Anne Johnston, Danielle Schneiderman, Rachel Kwan, Indira Thapa, Thomas E. Witte, Amanda Sproule, Steve Gleddie, Barbara Blackwell, David P. Overy

**Affiliations:** Ottawa Research & Development Centre, Agriculture & Agri-Food Canada, 960 Carling Ave., Ottawa, ON K1A 0C6, Canada; whynn.bosnich@agr.gc.ca (W.B.); anne.johnston@agr.gc.ca (A.J.); danielle.schneiderman@agr.gc.ca (D.S.); rachel.kwan@agr.gc.ca (R.K.); indira.thapa@agr.gc.ca (I.T.); tom.witte@agr.gc.ca (T.E.W.); amanda.sproule@agr.gc.ca (A.S.); steve.gleddie@agr.gc.ca (S.G.); barbara.blackwell@agr.gc.ca (B.B.)

**Keywords:** *Fusarium graminearum*, *Fusarium avenaceum*, species interaction, enniatins, beauvericin, ABC transporter

## Abstract

*Fusarium graminearum* and *F. avenaceum* often co-contaminate Canadian durum wheat grain, resulting in the co-deposition of species-specific mycotoxins, including trichothecenes produced by *F. graminearum* and enniatins produced by *F. avenaceum*. These mycotoxins pose significant risks to human and animal health. Although these fungi commonly co-occur in infected wheat, relatively little is known about how they interact during host infection. Interactions between the two species were examined using co-inoculation experiments on durum wheat spikes. In pathology trials, co-inoculations often reduced both disease severity and trichothecene accumulation compared with inoculations of *F. graminearum* alone, despite *F. graminearum* greatly out-competing *F. avenaceum* in total fungal biomass. Transcriptomic profiling identified strong induction of the *F. graminearum* ABC transporter gene *FgABCB8* during co-inoculation with an enniatin-producing *F. avenaceum* strain. When *F. graminearum* was grown in vitro, *FgABCB8* was induced upon exposure to *F. avenaceum* culture filtrate, or the related cyclohexadepsipeptides enniatin B1 or beauvericin. Heterologous expression of *FgABCB8* in yeast provided partial protection against enniatin and beauvericin toxicity. Gene disruption of *FgABCB8* increased *F. graminearum* sensitivity to enniatins. These findings demonstrate that *FgABCB8* expression enhances the ability of *F. graminearum* to tolerate enniatin-producing fungi.

## 1. Introduction

*Fusarium* cereal pathogens produce a diverse array of mycotoxins and other secondary metabolites that can contribute to plant disease development and, if consumed, have adverse effects on human and animal health. Fusarium Head Blight (FHB) is a globally significant cereal disease, reducing yield and grain quality and invoking food and feed safety issues due to mycotoxin deposition, especially the regulated mycotoxin deoxynivalenol. *F. graminearum* is the predominant pathogen influencing FHB severity in small grain cereals [1]. Although there has been progress towards introducing FHB resistance into bread wheat cultivars, equivalent improvements to commercial varieties of durum wheat have not occurred, and durum wheat is known to be quite susceptible to *Fusarium* infection [2]. While food processing can reduce mycotoxin levels, FHB-damaged durum grain can reduce pasta quality, and higher toxin concentrations in bran versus endosperm fractions mean that whole grain pasta products are at greater risk [3].

FHB-infected grain is often co-contaminated with numerous *Fusarium* species; *F. graminearum*, *F. poae*, *F. avenaceum*, and *F. sporotrichioides* are commonly isolated from Canadian wheat, barley, and oat samples [4,5,6]. *F. avenaceum* is pathogenic on a wide variety of crops, frequently infecting cereals, pulses, and canola [7,8], and thus crop rotation does not always compensate for minimal tillage consequences. Surveys of the *Fusarium* community in European grain samples indicated a high prevalence and co-occurrence of *F. graminearum* and *F. avenaceum* in sampled fields [9,10,11]. Environmental conditions strongly influence the abundance of *Fusarium* species; warm temperatures and rainfall favor *F. graminearum* infection while cooler temperatures are more conducive to *F. avenaceum* [12]. *F. avenaceum* has also been reported to be less sensitive than *F. graminearum* to fungicides applied to combat FHB, which may influence the *Fusarium* population dynamics in managed crop fields [13]. Infection of wheat spikes by *Fusarium* species quickly leads to the exclusion of other fungi, either through niche modification or competition between species [14]. Consequently, co-occurring *Fusarium* species within this exclusive disease complex must navigate intense interspecific competition that directly modulates the ultimate FHB phenotype, including disease severity and mycotoxin accumulation. To persist during co-infection, these prolific producers of secondary metabolite toxins have likely evolved specialized mechanisms to tolerate and cope with each other’s secreted metabolites.

Requiring significant energy inputs, the biosynthesis of secondary metabolites must provide fungi with an advantage, either to enhance host infection or protect the fungus from competition, predation, or other environmental stresses within its niche [15]. The genomes of *F. graminearum* and *F. avenaceum* contain a large number of secondary metabolite gene clusters, and each fungus produces a diverse array of secondary metabolites [16,17]. *F. graminearum* biosynthesizes trichothecenes such as 15-acetyldeoxynivalenol (15-ADON), 3-acetyldeoxynivalenol (3-ADON), or NX types, as well as the sesquiterpenoids culmorin, cyclonerodiol, and sambucinol, the meroterpenoid FDDPs, the polyketide zearalenone, and the nonribosomal peptide synthetase-derived gramillins A & B, fusaoctaxins (and associated cleavage products), and fusahexin [18,19,20,21]. Bioactive secondary metabolites produced by *F. avenaceum* include enniatins, moniliformin, acuminatopyrone, antibiotic Y, 2-AOD-3-ol, chlamydosporol, fusaoctaxins (and associated cleavage products), fusahexin, and JM-47 [16,22]. In addition, *F. avenaceum* and *F. graminearum* are both capable of producing aurofusarin (and associated biosynthesis intermediates), fusaristatin, butenolide, fusarins, and chrysogine [22]. A survey by the Canadian Grain Commission revealed that >70% of durum and spring wheat samples collected from 2010 to 2016 were contaminated with both DON and enniatins [23]. Italian durum pasta samples showed incidences of 100% and ≥90% for DON and enniatin B/A1, respectively [24]. Globally, cereal feed products are very commonly co-contaminated with multiple *Fusarium* mycotoxins [25,26].

Since *F. graminearum* and *F. avenaceum* and their mycotoxins often co-exist on cereals, we wanted to examine how these pathogens may compete for the same environmental niche. We investigated the interaction of *F. graminearum* and *F. avenaceum* on durum wheat and discovered an ABC transporter, FgABCB8, which is highly induced when *F. graminearum* shares an environmental niche with *F. avenaceum* or enniatins or beauvericin. The observed efflux activity of FgABCB8 implicates this transporter in providing *F. graminearum* with some protection against the toxicity of these cyclohexadepsipeptide mycotoxins.

## 2. Materials and Methods

### 2.1. Fungal Strains and Culture Conditions

Wild-type *F. graminearum* (*Fg*LH03 [DAOM242075] and *Fg*LH07 [DAOM242081]) and *F. avenaceum* (*Fa*LH03 [DAOM242076] and *Fa*LH27 [DAOM242378]) were originally isolated from Eastern Canadian red spring or winter wheat samples and provided by Dr. T. Grafenhan (Canadian Grain Commission, Winnipeg, MB, Canada). These strains are deposited in the Canadian Collection of Fungal Cultures (Agriculture & Agri-Food Canada, Ottawa, ON, Canada). *Fg*PH-1 (NRRL 31084) was kindly provided by Dr. K. O’Donnell (USDA/ARS/NCAUR, Peoria, IL, USA). Fungal cultures were initially grown on Synthetischer Nährstoffarmer agar (SNA) plates under an alternating 12 h, 25 °C light/12 h, 22 °C dark cycle. Spores of fungal cultures were stored long-term at −80 °C in 15% glycerol. Fungal growth assays on solid media were performed as previously described [18]. Macroconidial spores were generated by growth on half-strength potato dextrose agar (½PDA; BD Difco) plates for up to six days at 25 °C under fluorescent and black light to induce sporulation, and spores were collected as previously described [27].

### 2.2. Durum Wheat Inoculation

Durum wheat (*Triticum turgidum* L. var. durum) cultivar Langdon was grown as previously described [28]. Wheat head awns were trimmed and one spikelet per head point-inoculated at mid-anthesis with 1000 spores of *F. avenaceum* and/or 500 spores of *F. graminearum* in 5 µL of sterile water. Inoculated spikes were loosely covered with a plastic bag, and plants were misted for 1 min every hour for three days in a growth cabinet (25 °C/20 °C; 16 h photoperiod). After misting, plants remained in the growth cabinet with 80% humidity. Disease severity was assessed by counting the number of visually infected spikelets per inoculated spike at 5, 6, and 7 days post-inoculation (dpi), and then spikes were harvested and flash-frozen in liquid nitrogen. The experiment contained five independent replicates, each with four to six inoculated spikes per treatment. DON quantitation was done by ELISA using a DON-specific antibody, as previously described [29,30].

### 2.3. Species-Specific Fungal Biomass Quantification

Wheat heads were ground in a mortar and pestle in the presence of liquid nitrogen prior to extracting genomic DNA with an Illustra DNA Extraction Phytopure Kit (GE Healthcare, Mississauga, ON, Canada). Species-specific fungal biomass was quantified by droplet digital PCR (ddPCR) (reagents sourced from Bio-Rad Laboratories Canada Ltd., Mississauga, ON, Canada). *F. graminearum* and *F. avenaceum* species-specific primers and Taqman probes (Sigma-Genosys, Oakville, ON, Canada) were designed from the *FGSG_08823* (*FgABCB8*; FAM-labeled) and *Fa*LH03 Calmodulin (HEX-labeled) genes, respectively (Table S1). Approximately 5 ng of EcoRI-digested genomic DNA isolated from non-infected, singly inoculated, and co-inoculated durum wheat heads (4–6 heads pooled per biological replicate) were added to 25 µL ddPCR reactions using ddSupermix for Probes along with 640 nM of each primer and 250 nM of each probe in side-by-side duplicates, as well as no-template controls using a 57 °C annealing temperature and using the default cycling program for the ddProbe Supermix on the Bio-Rad C1000 Touch Thermal Cycler (Bio-Rad Laboratories Canada Ltd.). Durum wheat-specific Heterogeneous Nuclear Ribonucleoprotein Q (hnRNP Q) primers were used in ddPCR using the EvaGreen Supermix with a 58 °C annealing temperature using the default EvaGreen cycling program on the C1000 Touch Thermal Cycler (Bio-Rad Laboratories Canada Ltd.) to measure the amount of wheat DNA in the samples. Following amplification, the samples were read on the Bio-Rad QX200 Droplet Reader (Bio-Rad), and the technical replicates were combined. The species-specific amplified samples were then normalized against the hnRNP Q data. Fungal biomass was determined relative to wheat biomass within each replicate of pooled spikes.

### 2.4. Secondary Metabolite Profiling

*Fa*LH03 and *Fa*LH27 were each grown in 50 mL GYEP media [31] in 250 mL flasks with glass filters for 6 days (28 °C, 170 rpm in the dark), mycelia collected, rinsed and transferred to 50 mL of either formulation 1 (0.1 g (NH_4_)_2_HPO_4_, 0.3 g KH_2_PO_4_, 0.02 g MgSO_4_-7H_2_O, 0.5 g NaCl, 4 g sucrose, 1 g glycerol + 100 mL of distilled water) or formulation 2 (0.05 g (NH_4_)_2_HPO_4_, 3 mL 200 nM glutamine, 0.3 g KH_2_PO_4_, 0.02 g MgSO_4_-7H_2_O, 0.5 g NaCl, 4 g sucrose, 1 g glycerol + 97 mL of distilled water). After thirteen days, the culture supernatants were run through Bond Elute PLEXA columns (Agilent Technologies, Inc., Mississauga, ON, Canada), which had been pre-washed with 5% MeOH, and the 100% MeOH filtrate fractions were collected and pooled. The pooled *Fa*LH03/*Fa*LH27 MeOH fraction was lyophilized and stored at −20 °C.

To profile secondary metabolites, an aliquot of the pooled *Fa*LH03/*Fa*LH27 MeOH fraction was reconstituted 1:1 MeOH:H_2_O (500 µg/mL final concentration), filtered through a 0.45 µm PTFE filter, and a 5 µL aliquot was analyzed by UPLC-HRMS. Chromatography was performed using a Phenomenex Kinetex C_18_ 100 Å column (2.1 × 50 mm, 1.7 µm) with a flow rate of 0.35 mL/min. The mobile phase consisted of water containing 0.1% formic acid (solvent A) and acetonitrile containing 0.1% formic acid (solvent B). The gradient started at 5% solvent B and increased to 95% over 4.5 min, held at 95% for 3.5 min, returned to starting conditions over 0.5 min, and allowed to equilibrate for 5 min. The data was pre-processed using MZmine2 [32] and the *m*/*z* values in the resulting peak list were compared to an in-house database of *Fusarium* metabolites based on retention time and associated pseudomolecular ion masses (within 5 ppm mass tolerance). Chromatographic peaks with no database matches were annotated manually.

### 2.5. In Vitro Transcriptome Profiling

*Fg*LH03 macroconidia (8 × 10^4^ spores/mL) were used to inoculate 4 mL of GYEP media [31] in each well of 6-well plates. Sterile Miracloth circles were added to each well, plates sealed with air pore tape (Qiagen Inc., Montreal, QC, Canada), and incubated at 170 rpm at 28 °C in the dark. After two days, the media was removed and replaced with 4 mL of second-stage media [31]. At this time, either 10 µL of DMSO or 10 µL of mixed *Fa*LH03/*Fa*LH27 MeOH fraction re-suspended in DMSO was added to each well and incubated at 170 rpm, 28 °C in the dark. Miracloth circles and associated mycelia were harvested after 6 h, 1 day, and 2 days and flash-frozen in liquid nitrogen. RNA was extracted using an RNAeasy Plant & Fungal Mini Kit (Qiagen Inc.), according to the manufacturer’s instructions. RNA was quantified using a Nanodrop instrument (Thermo Scientific, Waltham, MA, USA). An aliquot was taken to view RNA quality on the Bioanalyzer using the corresponding RNA 6000 Nano Kit (Agilent Technologies Inc.). High quantity and quality RNA was kept at −80 °C until labeling.

RNA was labeled using the One-Color Quick Amp Labeling Kit and the One-Color Microarray-Based Gene Expression Analysis with Tecan HS Pro Protocol (Agilent Technologies Inc.), with the following modifications. A Robbins Scientific Model 400 Hybridization oven with an Agilent oven hybridization rotator G2530-60020 was used. The custom microarray slides (Agilent Technologies Inc., AMADID 020717) were 4 × 44K (4 arrays per slide, 44,000 oligos per array) with up to three individual 60-mer oligos representing each of 13,918 predicted *F. graminearum* genes. Statistical analysis (moderated *t*-test, adjusting limits on fold change) was performed on GeneSpring GX v11.0 software (Agilent Technologies Inc.) to determine genes of interest. False and low-intensity data points were removed from the data set by removing genes with intensities less than 50, and a moderated *t*-test removed genes with *p*-values greater than 0.05. The final data set was obtained by selecting those genes that had an absolute fold change of at least 3.0. Gene annotation was obtained at The Fungal and Oomycete Informatics Resources database (http://fungidb.org/, gene annotation confimation last verified 2 February 2026) [33]. Array data has been deposited at NCBI (GEO accession #GSE130915).

### 2.6. In Planta Transcriptome Profiling

Durum wheat heads were point-inoculated with single or dual species as described above and collected 4 days and 7 days post-inoculation. Each biological replicate was derived from the central eight spikelets of a single wheat head (4 biological replicates per treatment). RNA was isolated using a Qiagen RNeasy Plant Mini kit followed by a Qiagen DNAse treatment, according to the manufacturer’s instructions. RNA concentration and quality were verified using a QuickDrop spectrophotometer (Molecular Devices LLC., San Jose, CA, USA) and a 2100 BioAnalyzer (Agilent Technologies Inc.). RNAseq libraries were prepared using the Illumina NextSeq 500/550 High Output v2 kit, according to the manufacturer’s instructions, and the RNA sequencing was conducted as 150-nucleotide pair-end reads using Illumina HiSeq 2500 by the National Research Council Canada sequencing service (Saskatoon, SK, Canada). The RNASeq data files can be found in the NCBI BioProject PRJNA1423152.

Fastq read files were imported into CLC Genomics Workbench v20.0.4 and trimmed using default trimming parameters of adaptors and low quality sequences, discarding short reads < 60 nt. A combined genome genbank flat file was made consisting of the *F. graminearum* PH-1 genome (genbank accession GCF_000240135.3) merged with the de novo annotated *F. avenaceum* FaLH03 assembly [16]. *F. graminearum* gene models were simplified by reducing gene boundaries to the start/stop of respective coding sequences, overlapping genes were filtered by removal of the shorter gene model, and mRNA tracks were revised to match the CDS exon structures. Illumina RNAseq reads were mapped to the merged *F. graminearum* and *F. avenaceum* gene calls using stringent alignment parameters to improve species-specific read assignment: length fraction = 0.95 and similarity fraction = 0.95 (mismatch cost 2, insertion cost 3, deletion cost 3). Only uniquely mapping reads were retained for downstream expression analysis.

Gene-level expression was quantified in CLC using transcripts per million (TPM), as previously described [34]. Differential expression analysis was performed in CLC using pairwise comparisons between treatment and timepoint combinations, incorporating 4 biological replicates per treatment. Statistical significance was assessed using CLC’s built-in differential expression model with *p*-values corrected for multiple testing using the false discovery rate (FDR) method. Gene annotation was accessed within the FungiDB database [33].

### 2.7. Quantitative ddPCR

All RNA was extracted using an RNAeasy Plant & Fungal Mini Kit (Qiagen Inc.), according to the manufacturer’s instructions. For in vitro qPCR experiments, *F. graminearum* was grown in 6-well plates as for transcriptome profiling, with the following modifications. For the enniatin induction experiment, *Fg*LH03 was grown in GYEP media for two days, then in 2nd stage media [31] supplemented with either 10 µL of DMSO or 10 µL of 2 µg/mL enniatin B1 re-suspended in DMSO per well. To examine gene expression under nutrient-restricted or unrestricted conditions, *Fg*LH03 or *Fg*PH-1 was grown in GYEP media, then re-suspended in 4 mL of either CM (complete media), MMC (minimal media without a carbon source), or MMN (minimal media without a nitrogen source) [35]. The cultures were incubated at 140 rpm, 28 °C in the dark, harvested at 30 min and 2 h, and flash-frozen in liquid nitrogen. Frozen mycelia were ground in a mortar and pestle.

For *in planta* ddPCR assays, total RNA from at least three biological replicates was reverse transcribed using iScript Reverse Transcription Supermix for RT-PCR (Bio-Rad Laboratories Ltd.). Eight µL of cDNA or appropriate cDNA dilutions were used in 25 µL ddPCR reactions (two technical replicates) using EvaGreen Supermix with custom *F. graminearum FgABCB8* cDNA-specific primers (Sigma-Genosys) and the β-tubulin housekeeping gene (Table S1). Reactions were amplified with an annealing temperature of 59 °C using the default cycling program for EvaGreen Supermix on the C1000Touch Thermal Cycler. No template control ddPCR reactions were added for each set of primers. Following amplification, the samples were read on the QX200 Droplet Reader, and the technical replicates were combined. *FgABCB8* absolute values for each sample were normalized using the *β-tubulin* gene.

### 2.8. FgABCB8 Transcript Cloning and Protein Expression in Yeast

Construction of the FgABCB8 protein expression vectors is described in Supplemental Material: Supplementary Materials and Methods.

### 2.9. Western Blot Analysis

*S. cerevisiae* expressing the p416 N-terminal HA-tagged FgABCB8 was grown overnight in 5 mL of yeast peptone dextrose (YPD) media at 30 °C and 300 rpm. Yeast protein lysates were prepared as previously described [36]. Yeast lysate samples in 1X protein loading buffer (1 M Tris-HCL pH6.8, 10% SDS, 10% glycerol, 12.5% β-mercaptoethanol, and 1% Bromophenol blue) were heated at 55 °C for 5 min and loaded onto a 4–15% pre-cast Protein gel (Bio-Rad Laboratories Canada Ltd.) and subjected to 200 V for 45 min. The lysate was transferred to a 0.2 µm PDVF membrane using the Trans-Blot Turbo Transfer System according to the manufacturer’s instructions (Bio-Rad Laboratories Canada Ltd.). Antibody binding of HA-tagged proteins was done using the iBind Flex Western System (Invitrogen Canada Inc., Burlington, ON, Canada), according to the manufacturer’s instructions. The primary antibody used for detection was polyclonal Anti-HA antibody (Sigma-Aldrich, Oakville, ON, Canada) diluted 1:1000, and the secondary antibody was peroxidase-conjugated goat anti-rabbit (IgG; Cedarlane, Burlington, ON, Canada) diluted 1:10,000. Detection of the signal was accomplished using Clarity Max Western ECL blotting solutions (Bio-Rad Laboratories Canada Ltd.) according to the manufacturer’s instructions, and the image was captured using a ChemiDoc XRS+ System with Image Lab Software v6.0 (Bio-Rad Laboratories Canada Ltd.).

### 2.10. Fungal Growth Assays

Yeast microplate growth assays were conducted as previously described [36], with the following modifications. AD12345678 yeast expressing the p416-pdr5 N-terminal HA tagged *FgABCB8* vector (*FgABCB8_1*) or p416 vector alone (EV) as a control were inoculated in 5 mL of YPD media, and the microplate was incubated overnight at 300 rpm and 30 °C. Enniatin B (Sigma-Aldrich), beauvericin (Fermentek, Ltd., Jerusalem, Israel), culmorin, and 15-acetyldeoxynivalenol (isolated in-house and purity validated by NMR) were each re-suspended in DMSO. The growth rate of each strain was monitored by measuring the absorbance (600 nm) every 10 min over a 24 h period using a PowerWave XS2 plate reader (Biotek, Winooski, VT, USA).

*F. graminearum* microplate growth assays were conducted as previously described [37], with the following modifications. Wild-type and transgenic *F. graminearum* strains were inoculated (2500 spores into 200 µL potato dextrose broth [BD Difco]/well) into 384-well plates and grown with the addition of DMSO (<1%) as a control or 20 µg/mL enniatin A1, B, or B1 (Sigma) re-suspended in DMSO. The growth rate of each strain at 28 °C with slow continuous shaking was monitored over a 72 h period by measuring the absorbance (600 nm) every 2 h using an Eon microplate spectrophotometer (BioTek).

For all fungal growth assays, four biological replicates were performed, each with three technical replicates per treatment. Area under the curve (AUC) was calculated by integration of the growth curves using Gen5TM 3.11 (BioTek). Relative growth ratios (%) were determined by dividing the AUC of treated cells by the AUC of control cells.

### 2.11. Fusarium Gene Modification and Confirmation

*FgABCB8* was disrupted using the USER pRF-HU2 vector containing the hygromycin resistance gene (*hygR*) for selection, while complementation was done using the USER pRF-GU vector containing the geneticin resistance gene (nptII), via *Agrobacterium tumefaciens*, as previously described [38]. All PCR amplification was done with Advantage Taq (TakaraBio, San Jose, CA, USA). Primers were designed to amplify 1014 bp upstream (5′ region) and 852 bp downstream (3′ region) of the gene, including sequence overhangs (underlined sequence, Table S1) to facilitate cloning into pRF-HU2 or pRF-GU vectors. Selection of putative gene disruptant and complemented strains was conducted using 150 µg/mL of hygromycin or geneticin, respectively.

Putative transformants were grown on PDA, genomic DNA extracted using the E.Z.N.A. Fungal DNA Isolation Kit (Omega Bio-tek Inc., Norcross, GA, USA), and analyzed by PCR using gene-specific primers (Table S1). Single spores were isolated from all PCR-positive gene-disrupted and complemented strains. Southern analysis was performed using 10 µg of genomic DNA extracted with the Illustra DNA Extraction Kit (GE Health Care) and digested with *Nco*I (New England Biolabs, Whitby, ON, Canada). The hybridization probes (Figure S7) were generated using the PCR DIG labeling kit, and hybridizations were done using the DIG Detection Kit (Roche Diagnostics; distributed by Sigma-Aldrich, St. Louis, MO, USA), as per the manufacturer’s instructions.

To confirm the expression of the *FgABCB8* transcript, wild-type and transgenic strains were grown as outlined in Section 2.5, but with the addition of either DMSO (0.25%) or 2 µg/mL of enniatin B in DMSO to the media. Next, the fungal spores were grown in second-stage media for 2 h, and then total RNA was extracted as described in Section 2.5. Reverse transcriptase (RT)-PCR (Superscript IV, Invitrogen Canada Inc.) was performed using 2 µM of *FgABCB8*-specific reverse primer (Table S1), as per the manufacturer’s instructions, followed by PCR amplification (primers 20F and 20R) with final dNTP and primer concentrations of 0.2 mM and 0.2 µM, respectively. PCR products were visualized using SYBR Safe (Invitrogen Canada Inc.) stained 1% agarose gel. Transcript quantification was done using the ddPCR procedure as previously outlined in Section 2.6.

### 2.12. Yeast Enniatin Quantitation Assays

Overnight cultures of yeast AD1-8 expressing either *FgABCB8_1* or EV, grown in YPD at 300 rpm and 30 °C, were diluted to an OD (optical density at 600 nm) of 0.4 and grown under the same conditions for an additional 4 h. Yeast cultures were then inoculated in 1 mL YPD media at OD = 0.7 in the presence of DMSO (<1%; control) or 5 µg/mL of enniatin B re-suspended in DMSO. In addition, 1 mL of YPD media containing DMSO or 5 µg/mL enniatin B was incubated alongside the yeast as controls. At time 0, 3 h, or 6 h time points, aliquots were centrifuged and supernatant collected for detection of enniatin B by UPLC-HRMS. Chromatographic conditions were adapted to increase the separation of enniatin B and other mycotoxins using a Kinetex, F5 100 Ả, C18 column (Phenomenex, 2.1 × 50 mm, 1.7 µm) with a flow rate of 0.350 mL/min. Separation was achieved with mobile phase (A) being LC-MS grade water (Thermo Scientific) containing 0.1% formic acid and 5 mM ammonium acetate, and mobile phase (B) was LC-MS grade methanol (Thermo Scientific) containing 0.1% formic acid and 5 mM ammonium acetate. The gradient program started with 5% mobile phase B for 0.5 min, increased to 95% over the following 10.5 min, then held at 95% B for 3 min. Finally, the gradient returned to 5% B over the course of 2 min and remained at this concentration for 4 min to equilibrate between samplings. Enniatin B annotations were made by comparing retention times, accurate mass within 5 ppm, and mass fragmentation patterns observed from commercially purchased standards (Sigma Aldrich, Oakville, ON, CA). Under the above-specified conditions, enniatin B was eluted at 12.07 min. The ions detected were the pseudomolecular ions [M + NH_4_]^+^, [M + H]^+^.

Quantitation of enniatin B was carried out by building a processing method in Thermo XCalibur 2.2 software (Thermo Scientific). An extracted ion chromatogram of the precursor ion was generated using a ± 5 ppm mass accuracy threshold. The ICIS peak integration algorithm using 9 smoothing points and a baseline window of 70 was used. External calibration curves were used while building a processing method. The limit of detection (LOD) and limit of quantitation (LOQ) of the ion detected were determined as the lowest concentration achievable, whereupon the ion peak area of three consecutive injections resulted in a relative standard deviation (RSD) less than 20%.

Relative percent ratio was determined by calculating the percentage of the ratio of enniatin B concentration in yeast supernatant to enniatin B concentration in YPD media alone for each time point.

### 2.13. Statistical Analysis

Statistical analysis was performed using either one- or two-way ANOVA followed by Tukey Multiple comparison test (unless otherwise noted) in GraphPad Prism (GraphPad Software v 9, La Jolla, CA, USA).

### 2.14. UPLC-HRMS Operation Parameters

UPLC-HRMS profiling was performed using a Thermo Scientific Dionex Ultimate 3000 UHPLC system coupled to a Thermo LTQ Orbitrap XL high-resolution mass spectrometer (Thermo Scientific, Waltham, MA, USA). The HRMS was operated in ESI+ mode monitoring *m*/*z* 100–2000 using the following parameters: sheath gas (40), auxiliary gas (5), sweep gas (2), spray voltage (4.0 kV), capillary temperature (320 °C), capillary voltage (35 V), tube lens (100 V), maximum injection time (500 ms) and microscans (1).

## 3. Results

### 3.1. F. graminearum and F. avenaceum Co-Inoculation Often Leads to Reduced FHB Disease Phenotype and DON Contamination

**Figure 1:**
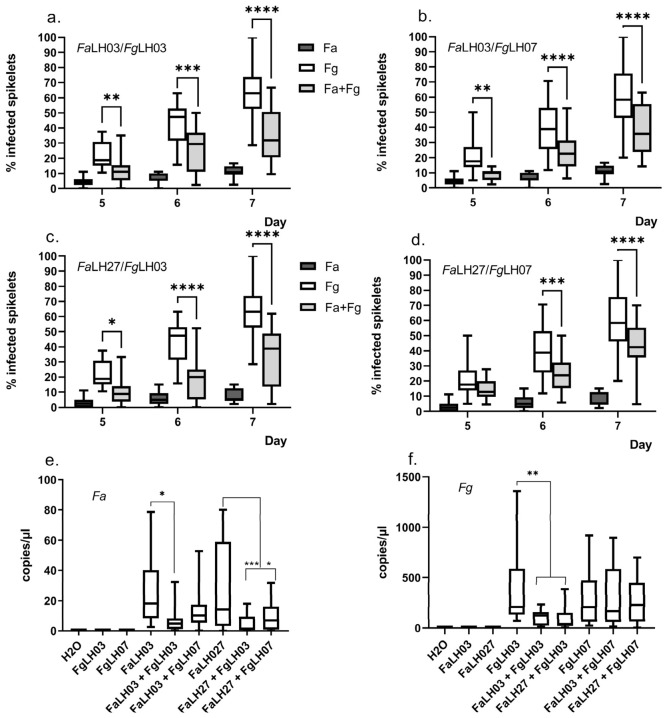
Impact of single or co-inoculation of *F. graminearum* and *F. avenaceum* on FHB disease development in durum wheat spikes. Data are presented as box-and-whisker plots, where the central horizontal line represents the median, the box boundaries indicate the first and third quartiles, and the whiskers denote the minimum and maximum values excluding outliers. (**a**–**d**). Visual disease assessment by percentage of infected spikelets, five to seven days post point inoculation (dpi) of four *F. graminearum*/*F. avenaceum* strain combinations (5 biological replications, 4–6 spikes per treatment replicate). (**e**,**f**). Fungal biomass using *Fusarium* species-specific primers (droplet digital PCR) for *F. graminearum* (**e**) and *F. avenaceum* (**f**) at 7 dpi after single, dual, or control (H_2_O) inoculations. * *p* < 0.05; ** *p* < 0.01; *** *p* < 0.005;**** *p* < 0.001.

Durum wheat spikes were inoculated with either a single strain of *F. avenaceum* (either *Fa*LH03 or *Fa*LH27) [16] or *F. graminearum* (*Fg*LH03, 3-ADON chemotype or *Fg*LH07, 15-ADON chemotype) as well as both species simultaneously. Disease symptoms were monitored up to seven days post-inoculation (dpi) because durum wheat is quite susceptible to *F. graminearum* [2], and wheat heads were severely infected after this time point. Infected spikelets showed brown discoloration, sometimes accompanied by mycelia visible on the surface. As expected, *F. graminearum* was much more pathogenic on durum wheat than *F. avenaceum*, which was reflected in the stronger disease symptoms and the 11 to 16-fold higher *F. graminearum* biomass relative to *F. avenaceum* in the single species inoculations (Figure 1). Co-inoculation led to significantly lower disease symptoms (% infected spikelets) in all four interspecies combinations by 6 dpi (Figure 1a–d) and in three combinations by 5 dpi, relative to *F. graminearum* alone. The co-inoculation of *Fg*LH03 with either *F. avenaceum* strain resulted in lower biomass of both fungal species in pooled spikes (Figure 1e,f) as compared to monoculture controls. Based on the observed reduction in disease symptoms and relative fungal biomass, *Fg*LH03 growth was more inhibited than *Fg*LH07 by the presence of *F. avenaceum*.

**Figure 2:**
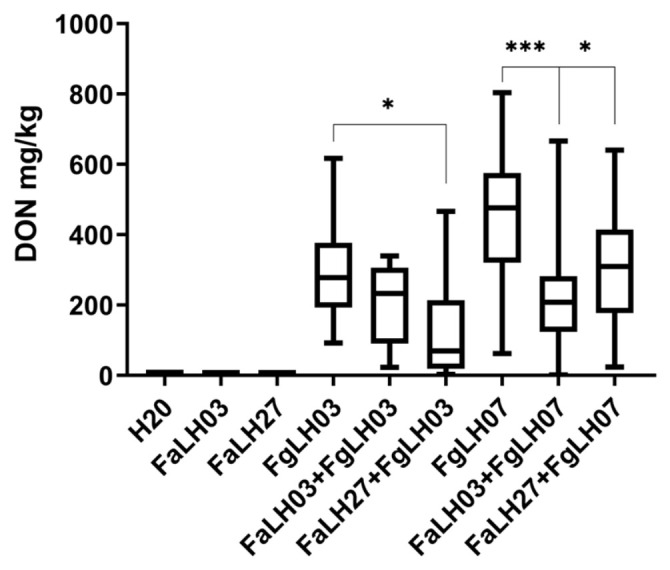
Deoxynivalenol (DON) concentration after single or co-inoculation of *F. graminearum* and *F. avenaceum* in durum spikes. Spikes were collected at 7 dpi. An inoculation control (H_2_O) is included. * *p* < 0.05; *** *p* < 0.005. Data are presented as box-and-whisker plots, where the central horizontal line represents the median, the box boundaries indicate the first and third quartiles, and the whiskers denote the minimum and maximum values excluding outliers.

Significantly lower DON levels were detected in three of four co-inoculation treatments relative to the respective *F. graminearum* inoculation alone (Figure 2). There was no significant difference in the ratio of DON accumulation to *F. graminearum* biomass between treatments, suggesting that the presence of *F. avenaceum* did not directly affect trichothecene production. Intriguingly, the co-inoculation of the two isolates originating from the same grain sample (*Fa*LH03, *Fg*LH03) led to DON levels not significantly different than *Fg*LH03 alone. As the DON-specific antibody used in the ELISA has been shown to recognize DON and 15-ADON but exhibits a lower binding specificity for 3-ADON [39], only DON levels involving the same *F. graminearum* chemotype were directly compared. Wheat cells can very efficiently de-acetylate 3-ADON and 15-ADON to DON [40]; the ELISA assay may fail to properly quantify any 3-ADON remaining in the tissues; however, 3-ADON levels are expected to be negligible.

### 3.2. Impact of F. avenaceum or Its Secondary Metabolites on the F. graminearum Transcriptome

Since *F. graminearum* continued to dominate during co-inoculations despite the potentially toxic array of secondary metabolites known to be excreted by *F. avenaceum*, the transcriptome of *F. graminearum* exposed to *F. avenaceum* secondary metabolites relative to no exposure was profiled. *F. avenaceum*-infected wheat heads harvested at maturity can contain, for example, enniatins, moniliformin, 2-aminodimethyloctadecanol, and chlamydosporol [41]. *Fa*LH03 and *Fa*LH27 were grown separately in liquid culture under conditions that induced the production of secondary metabolites. The secondary metabolites secreted into the culture supernatant were extracted, pooled, and profiled by UHPLC-HRMS. These mixed extracts were dominated by the presence of an array of enniatins, but ferricrocin, JM-47, and other unknown metabolites were also detected (Supplementary Figure S1 and Table S2). *F. graminearum* (*Fg*LH03) was grown in liquid culture to which the liquid medium was supplemented with either the extracted *F. avenaceum* secondary metabolome at a concentration equivalent to the original *F. avenaceum* culture (culture extract/culture volume), or with a blank extract suspension solvent (DMSO) used as a control. RNA extracted from at least three biological replicates of *F. graminearum* cultures at two time points after treatment (6 h and 2 d) was used to monitor the *F. graminearum* transcriptome.

Focusing on genes that exhibited a greater than three-fold change in expression between treatments (*p* <0.05), 60 up-regulated and 30 down-regulated genes were identified upon exposure to *F. avenaceum* culture filtrate relative to the control treatment (Supplementary Tables S3 and S4). Of the fifteen genes induced more than five-fold (Table 1), an ABC (ATP-binding cassette) transporter gene, *FgABCB8* (*FGRAMPH1_01G10921*, *aka FGSG_08823*) [42], was the most highly induced gene (>55-fold increase after 6 h).

To investigate the impact of *F. avenaceum* on the *F. graminearum* transcriptome in vivo, wheat heads were collected at 4 days and 7 days after inoculation with either *F. graminearum* (*Fg*LH03) or *F. avenaceum* (*Fa*LH03) or both species simultaneously. RNA sequencing reads were mapped to a merged *F. graminearum/F. avenaceum* reference genome database. Ten *F. graminearum* genes were significantly induced (FDR *p* < 0.001) at least three-fold in co-inoculated samples versus *F. graminearum* inoculated samples alone (Table S5), and three of these genes (*FGRAMPH1_01G010921*, *FGRAMPH1_01G21097*, *FGRAMPH1_01G04097*) were also observed as induced during in vitro exposure to *F. avenaceum* fungal filtrate. *FgABCB8* was the most highly upregulated, exhibiting 128-fold and 5-fold expression enhancement at 4 days and 7 days post-inoculation, respectively.

### 3.3. FgABCB8 Is Induced in the Presence of Enniatins

**Figure 3:**
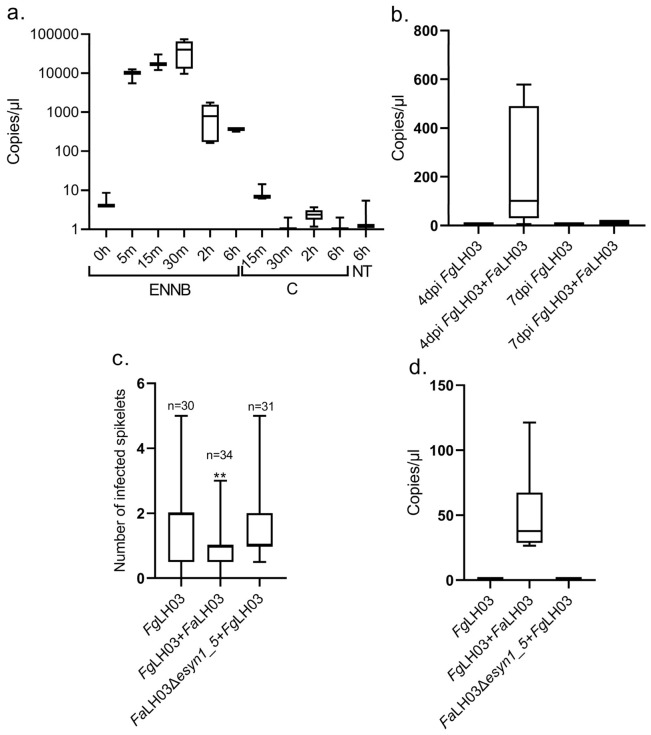
Expression of *FgABCB8* is induced in the presence of enniatins. Data are presented as box-and-whisker plots, where the central horizontal line represents the median, the box boundaries indicate the first and third quartiles, and the whiskers denote the minimum and maximum values excluding outliers. (**a**). *FgABCB8* expression (monitored by ddPCR) in *Fg*LH03 grown in vitro in the presence of enniatin B1 (ENNB, 10 µg/mL in DMSO) or DMSO alone (C, 2.5% DMSO) or no treatment (NT). Note the exponential scale of the *y*-axis. (**b**). *FgABCB8* expression (monitored by ddPCR) after single or dual species inoculation of durum wheat heads at four and seven days post-inoculation. (**c**). Box plots of FHB assays four days after inoculation of durum wheat spikes with *Fg*LH03 alone or together with *Fa*LH03 or *Fa*LH03∆*esyn1*_5.1 (four biological replicates). ** *p* < 0.01. (**d**). *FgABCB8* expression (monitored by ddPCR) after co-inoculation of durum wheat heads with enniatin-producing (*Fa*LH03) or enniatin-nonproducing (*Fa*LH03∆*esyn1_5.1*) *F. avenaceum* isolates at four days post-inoculation.

To examine the timing of *FgABCB8* induction more closely and test the induction ability of enniatin B1 (the most abundant *F. avenaceum* secondary metabolite in the mixed filtrate), *FgABCB8* expression was monitored by droplet digital PCR in 6-well plate cultures of *Fg*LH03, 5 min to 6 h after enniatin B addition (10 µg/mL) (Figure 3a). An approximate 1500-fold induction of *FgABCB8* was detected by 5 min after enniatin B1 addition, with a peak at 30 min; gene expression remained elevated 6 h after enniatin B1 exposure. *FgABCB8* is similarly induced by enniatin B in *Fg*LH07 under these conditionsn. In comparison, *FgABCB8* in *Fg*LH03, in the absence of enniatin B1, is expressed at very low levels in complete (CM), carbon-limiting (MMC), and nitrogen-limiting (MMN) media, as is *Fg*ABCB8 in the reference strain *Fg*PH1 (Figure S2).

ddPCR was also used to confirm that *FgABCB8* is strongly induced during wheat head infection, but only when *F. graminearum* is co-inoculated with *F. avenaceum* (Figure 3b). The *FgABCB8* primers do not amplify any sequences in *Fa*LH03 under the experimental conditions.

Previously constructed deletion mutants of the *F. avenaceum* enniatin synthase gene (*Fa*LH03∆e*syn1*) could no longer produce enniatins but remained as aggressive in infecting durum wheat as the progenitor wild-type strain [28]. Durum wheat spikes were inoculated with either *Fg*LH03 alone or *Fg*LH03 co-inoculated with the wild-type *Fa*LH03 or a *Fa*LH03∆e*syn1* mutant. At 4 dpi, the number of infected spikelets (brown discoloration) in the *Fg*LH03/*Fa*LH03 co-inoculated spikes was significantly less than that of *F. graminearum* alone (Figure 3c). However, co-inoculating *Fg*LH03 with the enniatin-nonproducing *F. avenaceum* isolate resulted in similar FHB symptoms to those observed with *Fg*LH03 in wheat heads without competition at 4 dpi. By 7 dpi, there was no significant difference between the co-inoculations of *Fg*LH03/*Fa*LH03 and *Fg*LH03/*Fa*LH03∆e*syn1*. Monitoring gene expression in these inoculated spikelets at 4 dpi revealed that *FgABCB8* is only induced during co-inoculation of durum spikes with wild-type *Fa*LH03 and not an enniatin-nonproducing mutant (*Fa*LH03∆e*syn1*) (Figure 3d). This suggests *F. graminearum* quickly responds by producing the FgABCB8 transporter as a defense against enniatin toxicity.

### 3.4. FgABCB8 Expression in Yeast Reduces Enniatin Toxicity

**Figure 4:**
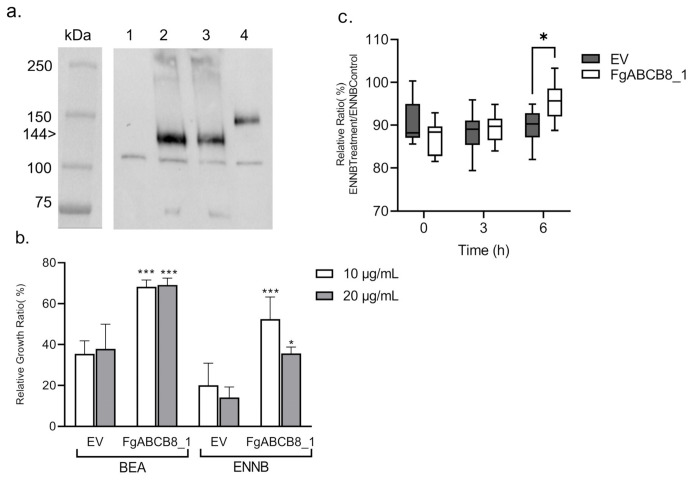
Expression of FgABCB8 protects *S. cerevisiae* AD12345678 from the growth-inhibiting effects of beauvericin and enniatin B1. (**a**). Western blot analysis demonstrating expression of FgABCB8 in yeast. FgABCB8 was detected using an antibody directed against the N-terminal HA tag. Lane 1, AD12345678_EV (empty vector control); lane 2, AD12345678_HA_PDR5promoter*FgABCB8*_1; lane 3, AD12345678_HA_PDR5promoter*FgABCB8*_2; lane 4, Δpdr5_HA-pdr5promoter-*pdr5* (positive control for HAtag). (**b**). Growth inhibition of *S. cerevisiae* AD12345678 transformed with either an empty vector (EV) or *FgABCB8* driven by the *PDR5* promoter in the presence of 10 or 20 µg/mL beauvericin (BEA) and enniatin B1 (ENNB). Relative growth ratios (%) were determined by dividing the area under the curve (AUC) of treated yeast cells by the AUC of untreated (DMSO) yeast cells of the same genotype (* *p* < 0.05; *** *p* < 0.005; bars represent standard deviation). (**c**). The relative ratio of enniatin B concentration in culture supernatant increases over time with the expression of FgABCB8. Relative percent ratio was determined by dividing the enniatin B concentration in yeast supernatant (ENNB treatment) by the enniatin B concentration in YPD media alone (ENNB Control) for each time point (* *p* < 0.05). Starting enniatin B concentration is 5 µg/mL. Data are presented as box-and-whisker plots, where the central horizontal line represents the median, the box boundaries indicate the first and third quartiles, and the whiskers denote the minimum and maximum values excluding outliers.

The FgABCB8 protein was expressed in *Saccharomyces cerevisiae* to test its properties in the presence of mycotoxins. *S. cerevisiae* is quite resistant to toxic metabolites, and consequently, strains (e.g., AD12345678) with multiple deletions of PDR drug efflux transporters have been constructed to facilitate screening for novel fungicides and determine transporter substrate specificity [43]. To express FgABCB8 in *S. cerevisiae* AD12345678, vectors were constructed with a hemagglutinin (HA) epitope tag linked to the 5′ end of the *FgABCB8* transcript, using either one of two different promoter/terminator combinations to drive expression, the native *S. cerevisiae PDR5* promoter and the weaker *CYC1* promoter, and their associated terminators (Figure S3). The ABCG family transporter PDR5p is capable of exporting a broad range of structurally diverse molecules of a similar range of molecular size, which includes DON [36,44]. By screening for the HA-tag, expression of FgABCB8 (144 kDa band) driven by the *PDR5* promoter was detected (Figure 4a); potential FgABCB8 expression driven by the *CYC1* promoter was not detectable by Western analysis.

Liquid growth assays of *S. cerevisiae* AD12345678 transformed with either an empty vector (EV) or expressing FgABCB8 were conducted in the presence of various mycotoxins. Under control conditions, there is no significant growth difference between *Sc*AD12345678 transformed with an empty vector or expressing FgABCB8 (Supplementary Figure S4a–c). However, in the presence of 10 µM enniatin B1, or the closely related cyclohexadepsipeptide mycotoxin beauvericin, growth of *Sc*AD12345678 is significantly reduced unless the yeast is expressing FgABCB8 (Figure 4b; Supplementary Figure S4). Staining to determine yeast viability showed no differences in viability between treatments. While the stronger *PDR5* promoter constructs are more effective, even constructs containing the weaker *CYC1* promoter driving *FgABCB8* provide some protection against growth inhibition (Supplementary Figure S4c,d). FgABCB8 expression does not provide any growth advantage in the presence of other *F. graminearum* mycotoxins, namely culmorin, and 15-ADON (Supplementary Figure S5).

The ability of yeast expressing FgABCB8 to export enniatins was monitored using UPLC-HRMS to determine enniatin B concentration in yeast culture supernatant (Supplementary Figure S6). Six hours after spiking the cultures with 5 µg/mL enniatin B, significantly more enniatin B was detected in culture filtrate when the yeast was expressing FgABCB8 compared to the empty vector (Figure 4c). The assay was limited to 6 h because during this time interval, no significant difference in growth was observed between the yeast strain expressing FgABCB8 and the yeast strain containing the empty vector.

### 3.5. FgABCB8 Gene Deletion Increases Enniatin Susceptibility

**Figure 5:**
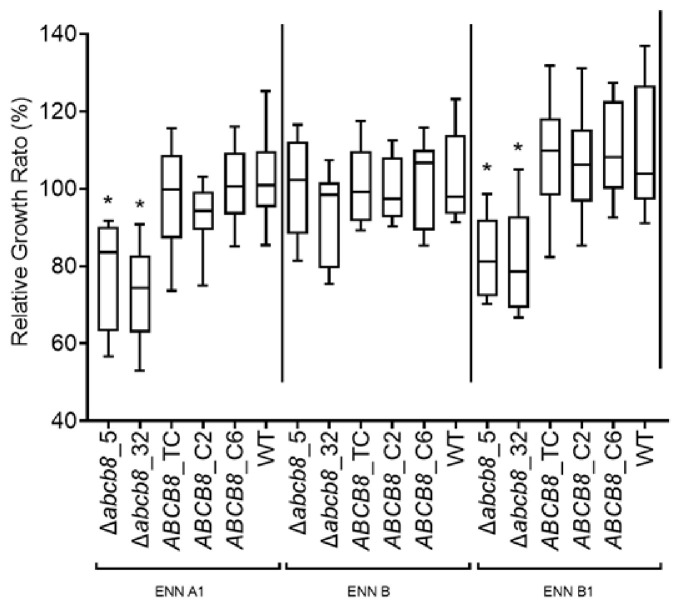
Loss of *FgABCB8* leads to increased enniatin toxicity to *F. graminearum*. Growth assay of two gene disruption mutants (∆*abcb8*), two gene complementation mutants (*ABCB8*_C), a transformant control (*ABCB8*_TC), and wild-type (WT, *Fg*LH03) with either added DMSO (<1%) or one of three enniatins (enniatin A1, enniatin B, and enniatin B1) at 20 µg/mL dissolved in DMSO over 72 h in 384-well microplates. Relative growth ratios (%) were determined by dividing the AUC of treated cells by the AUC of control (DMSO) cells. * *p* < 0.05. Four technical replicates per treatment were conducted in each of three biological replicates. Data are presented as box-and-whisker plots, where the central horizontal line represents the median, the box boundaries indicate the first and third quartiles, and the whiskers denote the minimum and maximum values excluding outliers.

To investigate the impact of the loss of *Fg*ABCB8 in *F. graminearum*, three independent *FgABCB8* deletion mutants (∆*abcb8*) were constructed in the *Fg*LH03 wild-type background (Supplementary Figure S7). One of the disruption mutants (∆*abcb8_5*) was complemented with a full-length *FgABCB8* gene, which included its native promoter (six independent strains, e.g., *ABCB8*_C2) (Supplementary Figure S8). *FgABCB8* expression was shown to be inducible by enniatin in complemented strains similar to wild-type by ddPCR (Supplementary Figure S8). Growth rate assays on solid PDA media indicated no differences between wild-type and transgenic strains, while slight growth differences were observed on minimal media (Supplementary Figure S9). The spore germination and growth of wild-type and transgenic strains were monitored in the presence of three enniatins (enniatin A1, B, and B1) (Figure 5). At 20 µg/mL, enniatin A1 and B1 significantly inhibited (*p* < 0.0001) *F. graminearum* lacking *FgABCB8,* while *ABCB8*-complemented and transformation control strains did not behave differently from wild-type. Enniatin B at this concentration did not noticeably impact the growth of any of the strains. Enniatin B has the lowest antimicrobial activity against assorted fungi, including *F. graminearum*, and Gram-positive bacteria, compared to the other enniatins [45]. This research supports the hypothesis that FgABCB8 helps protect *F. graminearum* from the toxicity of enniatins.

## 4. Discussion

The ability of mycotoxigenic fungi to compete is mainly dependent on their growth rate, production/tolerance of toxic metabolites, and ability to respond to environmental conditions [46]. Most *Fusarium* interspecific interaction studies also observed competitive interactions between species [47,48,49,50]. Co-inoculations of different *Fusarium* species often result in lower trichothecene content in grain, but when species-specific fungal biomass is considered, different studies have found either unaffected [51,52,53] or increased trichothecene production [47,48]. Ederli et al. reported that in vitro exposure of *F. avenaceum* to DON alone led to increased growth [54]. *Fusarium* species have also been observed to exhibit resistance to mycotoxins produced by other *Fusaria* [55]. *F. avenaceum* and *F. poae* are considered to be less aggressive pathogens relative to *F. graminearum* in the FHB complex [27,56]. Tan et al. concluded that wheat co-inoculated with *F. graminearum* and *F. poae* led to higher *F. poae* biomass than *F. poae* inoculated alone and proposed that *F. poae* benefited from the cell injury accompanying *F. graminearum* infection [53]. In contrast, we observed reduced *F. avenaceum* biomass when co-inoculated with *F. graminearum, s*uggesting that *F. avenaceum* is inhibited by the presence of the more aggressive pathogen. This dynamic likely reflects broader ecological trends where dominant pathogens rapidly colonize host tissues and lower overall endophytic fungal diversity [14], either outcompeting the community directly or hindering it by modifying the internal environment of the kernel. The observed reduction in infection symptoms, *F. graminearum* biomass, and DON accumulation during co-inoculation highlights how competitive microbial interactions within the spike can significantly modulate both FHB severity and mycotoxin contamination dynamics *in planta* under pathogen challenge. Moreover, particular species of endophytic fungi have been found to co-occur in asymptomatic kernels infected with *F. graminearum*, thus leading researchers to propose a role of naturally occurring endophytes to act as potential biological control agents that outcompete or prevent FHB in wheat [14].

ABC transporter proteins are involved in the ATP-dependent transport of a broad range of substrates across the membrane [57]. In fungal pathogens, ABC transporters are involved in pheromone and mycotoxin secretion and provide protection against toxic compounds such as phytoalexins and fungicides [58]. *FgABCB8* was strongly induced during *F. graminearum*/*F. avenaceum* interactions *in planta* or exposure to enniatins *in vitro*. Ipcho and associates had observed that *FgABCB8* is weakly induced by 1 h after application of the bacterial MAMP (microbe-associated molecular pattern) peptidoglycan [59]. *FgABCB8* encodes a member of the ABCB2 transport (also known as MDR, multidrug resistance) of full-length ABC-B proteins, with two nucleotide-binding domains (NBDs) and two transmembrane domains (TMDs) [42,57]. The best studied member of the ABCB2 subfamily is the human P-glycoprotein (ABCB1/MDR1), which is a highly promiscuous multidrug efflux transporter documented to transport over 300 compounds and involved in drug resistance in cancer cells [60]. The rapid induction of *FgABCB8* expression in the presence of enniatins is reminiscent of the induction of the *S. cerevisiae* PDR5 transporter by xenobiotic compounds [61]. The *F. graminearum* genome contains nine ABCB2 members [42]. *FgABCB8* was one of 60 *F. graminearum* ABC transporter genes disrupted by Yin and associates [62]; they observed no or minimal impact on wheat spike virulence when any of the full-length ABCB2 genes were disrupted. Other fungal ABCB2 proteins include the *Aspergillus nidulans* transporter AtrC, which is induced by cycloheximide [63], *Tricophyton rubrum* TruMDR5, which is an efflux pump for itraconazole [64], and ABC3 of *Magnaporthe oryzae*, which is involved in the efflux of a digoxin-like steroidal glycoside expressed during appressorial host penetration [65,66]. The most closely related, functionally characterized ABCB2 transporter to FgABCB8 is Abc3 in *Neurospora crassa*, a transporter of the drug staurosporine [67].

An epoxide hydrolase (*FGRAMPH1_01G21097*) and a sulfoxide synthase (*FGRAMPH1_01G04097*) were induced in *F. graminearum* in the presence of *F. avenaceum* secondary metabolites either *in vitro* or *in planta*. These enzymes could possibly be involved in the modification or detoxification of enniatins or other *F. avenaceum* metabolites. Two MFS (Major Facilitator Superfamily) multidrug transporters (*FGRAMPH1_01G17151, FGRAMPH1_01G03861*) were also up-regulated approximately seven-fold after two days of exposure to *F. avenaceum* filtrate in liquid culture. FGRAMPH1_01G03861 is related to vacuolar amino acid transporters in yeast, fnx1 and fnx2. *FgABCG6* (*FGRAMPH1_01G15627*, also known as *ABC1/FgABC3* [42,68,69]) was slightly up-regulated two to three-fold (Table S3). Five genes within the fusarin biosynthetic gene cluster (*FUS3*, *FUS6*, *FUS7*, *FUS8*, and *FUS9* [70]) were induced three to five-fold. The fusarin gene cluster is induced under acidic, high nitrogen conditions [71]. Two of the transporters, *FGRAMPH1_01G03861* and *FUS6*, have also been observed to be induced after a 6 h exposure to the fungicide trifloxystrobin as well as DON in liquid culture [72]. Induced over six-fold in the presence of *F. avenaceum* filtrate, *FGRAMPH1_01G08415* has been identified as a strong effector candidate [73].

Enniatins and beauvericin are cyclohexadepsipeptides made up of alternating hydroxyisovaleric amino acids and 3 N-methylated amino acids. These mycotoxins are among the most commonly found to contaminate cereal samples [23,74]. The lipophilic structure of these cyclohexadepsipeptides facilitates incorporation into membranes and the formation of ion channels, affecting ionic homeostasis [75,76]. In mammalian cell lines, enniatins promote potassium ion influx into mitochondria, leading to mitochondrial swelling and damage [77]. Enniatins and beauvericin have been reported to exhibit antimicrobial and fungicidal activity [45,78]. Enniatins have been shown to bind to and inhibit the multidrug transporter of yeast PDR5 (ABCG) and human ABCB1 and ABCG2 [79,80,81]. Tong and associates demonstrated that beauvericin can compete for the internal cavity of fungal multidrug ABC transporters to directly interfere with antifungal drug export [82]. Thus, the presence of this class of mycotoxins may inhibit the efflux and increase the toxicity of co-occurring toxic compounds.

Representatives from several *Fusarium* species complexes produce enniatins or the related beauvericin, including *Sambucinum* (e.g., *F. poae*, *F. sporotrichioides*), *Tricinctum* (e.g., *F. avenaceum*, *F. acuminatum*), and *Incarnatum-equiseti* (e.g., *F. equiseti*), and all of the above-named species are members of the FHB complex infecting cereals along with *F. graminearum* [5,83]. FgABCB8 orthologues can be identified in closely related species that are not cyclohexadepsipeptide producers, such as *F. culmorum* (96% amino acid identity) and *F. pseudograminearum* (95% amino acid identity) (Supplementary Figure S10). Beauvericin or enniatin producers of the *Sambucinum* (*F. sporotrichioides*, *F. poae*, *F. langsethiae*), *Tricinctum* (*F. avenaceum*), and *Fujikuroi* (*F. verticillioides*) species complexes also house putative FgABCB8 orthologues (77–89% amino acid identity) which are unlinked to their respective beauvericin/enniatin biosynthetic gene clusters. Enniatins have previously been shown to inhibit growth of *F. graminearum* *in vitro* (minimum inhibitory concentration 25 to 50 µM) and have been suggested to confer an advantage to *F. avenaceum* during competition with *F. graminearum* [45,54]. *F. graminearum* is not capable of biosynthesizing either enniatins or beauvericin but does produce other phytotoxic cyclic peptides known as gramillins, which can also target and disrupt plant cellular membranes [18,84]. Since enniatins or beauvericins are produced by such a wide variety of *Fusaria*, the ability to tolerate the presence of cyclohexadepsipeptides would be beneficial to those *Fusarium* species not producing this class of mycotoxins but often co-existing in the same environmental niches. This prolonged coexistence within multi-species disease complexes has likely exerted strong selective and co-evolutionary adaptive pressures, driving the utilization of specialized mechanisms like FgABCB8-mediated tolerance to withstand competitor-produced mycotoxins.

## 5. Conclusions

A *F. graminearum* ABC transporter gene, *FgABCB8*, is induced in the presence of enniatins and provides protection against enniatin toxicity. The classification of FgABCB8 as an MDR-type ABC transporter and the detection of higher enniatin concentrations in FgABCB8-expressing yeast culture filtrates suggest that enniatin tolerance is achieved through FgABCB8-mediated efflux. FgABCB8-mediated tolerance to enniatins contributes to the ability of *F. graminearum* to tolerate the presence of *F. avenaceum* during species interactions.

## Figures and Tables

**Table 1:** *F. graminearum* (*Fg*) genes up-regulated >five-fold (*p* < 0.05) after six hours and/or two days exposure to *F. avenaceum* (*Fa*) culture filtrate compared to the control (C) in liquid culture. FC, fold change.

FungiDB Gene Model	Mean FC, 6 h	Mean FC, 2 d	Product Description
*FGRAMPH1_01G10921*	55.34	411.70	FgABCB8, ABC transporter
*FGRAMPH1_01G06841*	1.40	8.77	hypothetical protein
*FGRAMPH1_01G13447*	3.79	8.11	hypothetical protein
*FGRAMPH1_01G17151*	1.34	7.66	MFS multidrug transporter TCR1
*FGRAMPH1_01G03861*	2.19	7.57	Multidrug resistance fnx1
*FGRAMPH1_01G04873*	1.72	7.07	Unspecified product
*FGRAMPH1_01G08415*	1.48	6.38	Glycosyl hydrolase family 18-6
*FGRAMPH1_01G08519*	6.37	1.34	short-chain dehydrogenase reductase family
*FGRAMPH1_01G02235*	−1.30	5.94	hypothetical protein
*FGRAMPH1_01G20953*	−1.03	5.50	Polyketide synthase
*FGRAMPH1_01G25707*	2.96	5.33	FUS9, methyltransferase
*FGRAMPH1_01G11935*	1.02	5.16	Beta-lactamase family
*FGRAMPH1_01G00403*	2.50	5.12	Zc053, transcription factor
*FGRAMPH1_01G04919*	1.13	5.12	hypothetical protein
*FGRAMPH1_01G21331*	1.37	5.01	C6 finger domain

## Data Availability

Access to all fungal strains used/generated in this research can be obtained through Agriculture & Agri-Food Canada via consultation with the corresponding author or by contacting the Canadian Collection of Fungal Cultures (AAFC, Ottawa, ON, Canada). In planta RNAseq datafiles can be found in the NCBI BioProject PRJNA1423152. In vitro microarray data has been deposited at NCBI (GEO accession #GSE130915).

## Supplementary Materials

The following supporting information can be downloaded at: https://www.mdpi.com/article/10.3390/jof12070524/s1, Figure S1: UHPLC-HRMS total ion current (TIC) chromatogram of the mixed methanol extracts of *Fusarium avenaceum* LH27 and LH03; Figure S2: *FgABCB8* gene expression in wildtype strains *Fg*LH03 and *Fg*PH1 during growth in complete (CM), carbon limiting (MMC) and nitrogen limiting (MMN) liquid media; Figure S3: Protein expression vector constructs for use in yeast; Figure S4: Expression of *FgABCB8* protects yeast from the growth-inhibiting effects of enniatin B1 and beauvericin; Figure S5: FgABCB8 expression does not protect yeast against culmorin or 15-acetyl-deoxynivalenol; Figure S6: LCMS analysis method of enniatin B; Figure S7: Structure and Southern confirmation of *FgABCB8* gene disruption; Figure S8: Validation of *FgABCB8* complemented strains; Figure S9: Solid growth assay of wildtype and derived transformants; Figure S10: Jukes-Cantor/Neighbor-joining consensus tree of Clustal Omega alignment of FgABCB8 orthologues in representative *Fusarium* cereal pathogens; Table S1: List of primers; Table S2: Metabolites detected in the mixed methanol extracts of *F. avenaceum* LH27 and LH03; Table S3: *F. graminearum* genes (60) up-regulated >three-fold in the presence of *F. avenaceum* culture filtrate versus DMSO control (*p* < 0.05); Table S4: *F. graminearum* genes (30) down-regulated >three-fold in the presence of *F. avenaceum* culture filtrate versus DMSO control (*p* < 0.05); Table S5: RNASeq analysis revealed ten *F. graminearum* genes induced greater than three-fold (FDR *p* < 0.001) at 4 or 7 days post *F. graminearum/F. avenaceum* co-inoculation vs. *F. graminearum*-inoculation of durum wheat heads.

## Abbreviations

The following abbreviations are used in this manuscript:

FHB	Fusarium Head Blight
DON	Deoxynivalenol
ABC	ATP-binding cassette
dpi	Days post-inoculation
