# Supplementary material for: An ABC-B Transporter Helps Protect Fusarium graminearum Against Enniatin Toxicity
Source: J Fungi (Basel). 2026 Jul 17;12(7):524. doi: 10.3390/jof12070524 (PMC13412724; doi:10.3390/jof12070524)
Supplement: Supplementary file 1 [file jof-12-00524-s001.zip › Supplementary Materials - SI Methods_Figures S1-S10.pdf]

Supplementary Materials

An ABC-B Transporter Helps Protect *Fusarium graminearum* Against Enniatin Toxicity

Linda J. Harris *, Whynn Bosnich, Anne Johnston, Danielle Schneiderman, Rachel Kwan, Indira Thapa, Thomas E. Witte , Amanda Sproule, Steve Gleddie, Barbara Blackwell and David P. Overy *

Ottawa Research & Development Centre, 960 Carling Ave., Agriculture & Agri-Food Canada, Ottawa, ON, Canada K1A 0C6; Ottawa Research & Development Centre, Agriculture & Agri-Food Canada, 960 Carling Ave., Ottawa, ON K1A 0C6, Canada; whynn.bosnich@agr.gc.ca (W.B.); anne.johnston@agr.gc.ca (A.J.); danielle.schneiderman@agr.gc.ca (D.S.); rachel.kwan@agr.gc.ca (R.K.); indira.thapa@agr.gc.ca (I.T.); tom.witte@agr.gc.ca (T.E.W.); amanda.sproule@agr.gc.ca (A.S.); steve.gleddie@agr.gc.ca (S.G.); barbara.blackwell@agr.gc.ca (B.B.)

* Correspondence: linda.harris@agr.gc.ca (L.J.H.); david.overy@agr.gc.ca (D.P.O.)

Supplemental Materials and Methods:

FgABCB8 transcript cloning and protein expression in yeast

All amplified PCR products were gel purified using the Qiagen Gel Purification kit (Qiagen, Montreal, ON, CA) and all ligations were done using T4 DNA ligase (Promega, Madison, WI, USA) according to manufacturer's instructions. All plasmid transformations were done using OneShot Top 10 electrocompetent cells (Invitrogen, Burlington, ON, CA) and plasmids isolated using the Qiagen plasmid extraction kit (Qiagen).

Using the Qiagen Plant RNA extraction kit (Qiagen), total RNA was extracted from 100 mg of *F. graminearum* mycelia (DAOM242081) which had been grown in the presence of Enniatin B (2 µg/mL) (Sigma-Aldrich, Oakville, ON, CA) for fifteen minutes. The cDNA of FgABCB8 was isolated using the FirstChoice RLM Race Kit (Ambion, Austin, TX, USA) and 5′ and 3′ Rapid Amplification of cDNA ends (RACE (Rapid Amplification of cDNA Ends)) was performed as outlined in the manual. Advantage 2 Polymerase (TakaraBio, Mississauga, ON, CA) was used for all polymerase chain reaction (PCR) amplifications according to manufacturer's instructions, using final dNTP and primer concentrations of 0.2 mM and 0.2 µM, respectively. A total of 9 µg and 0.9 µg of total RNA was used for 5′ and 3′ RACE, respectively. The gene-specific reverse primer was used for the reverse transcription reaction of the 5′ RACE (Table S1). Amplification of the cDNA:RNA duplex was accomplished using the 5′ outer primer
(supplied with the kit) and the reverse GSP1057R followed with an additional nested PCR using 5′ inner primer (supplied with kit) and nested reverse GSP1012R to yield a

1.1 kb fragment (5′ RACE product). Cycling conditions were 94 °C: 3 min, 94 °C: 30 sec, 60 °C: 30 sec, extension: 72 °C: 3 min, 35 cycles, final extension 72 °C: 7 min for all PCR amplification reactions. The 3'RACE adapter (supplied with the kit) was used for the reverse transcription reaction, followed by PCR using the supplied 3' outer primer with the forward GSP2221F (cycling conditions: 94 °C: 3 min, 94 °C: 30 sec, 65 °C: 30 sec, 72 °C: 3 min with a final extension of 72 °C: 7 min, 35 cycles). A nested PCR was done using 3' inner primer and GSP1248F, using the same cycling conditions to yield a 2.7 kb fragment (3'RACE product). A further 3' RACE reaction was performed using the reverse GSP2214R followed by PCR with GSP86F. Cycling conditions: 94 °C: 3:00 min, 94 °C: 30 sec, 66 °C:30 sec, 72 °C:3 min with a final extension of 72 °C:7 min yielding a 2.2 kb DNA fragment to close any gaps in the cDNA. This fragment was then digested using BamHI/NotI to produce an 844 bp fragment. To ligate all three cDNA fragments together, the 5' and 3' inner primer supplied with the kit were modified to contain the restriction sites EcoRI and SacII respectively. The 1.1 kb 5'RACE product was digested using restriction sites EcoRI/BamHI. The fragment was then ligated into pBluescript SK+ EcoRI/BamHI (Agilent Technologies Inc.). The construct was digested (BamHI/NotI) and ligated with the 844 bp cDNA fragment (1317/1279; BamHI/NotI). The resulting clone (4.9Kb) was digested using NotI/SacII and ligated to the 2.7 kb 3'RACE product (NotI/SacII). The final pBluescript SK+(3kb)_cDNAFgABCB8 (4.1 kb) was verified by sequencing.

The yeast expression vector p416-CYC (DualSystems Biotech, Zurich, Switzerland) was used to generate an N terminal HA-tagged *FgABCB8* expression vector, flanked by either a strong promoter and associated terminator (PDR5) or a weaker promoter and associated terminator (CYC) (Fig S1). All PCR amplification conditions using Pfu polyermase (TakaraBio, Mississauga, ON, CA) used reaction volumes of 50 μL with final concentrations of dNTP and primers of 0.2 mM and 0.2 μM, respectively. To generate the p416CYC expression vector with the pdr5 promoter/terminator, the vector was digested with XbaI/SacI to remove the CYC promoter and terminator (4.8 kb). The plasmid pBluescript SK+(3 kb) _cDNAFgABCB8 (3792 bp) was used as a template (10 ng) for amplification using primers (Table S1) to introduce a partial HA tag to the N terminal region. PCR product (3.8 Kb) was purified and used as a template (0.2 ng) for amplification with primers (HA1F and GSP3817R; Table S1) to incorporate the HA tag (45 bp). The PCR fragment was digested with XbaI to generate a 3770 bp fragment. The PDR5 promoter (594 bp) and terminator (305 bp)

were cloned using the pPDR5-416 plasmid as a template to amplify the pdr5 promoter and the pdr5 terminator (Table S1). A series of amplifications led to incorporate the 32 bp of the 3′ end of the *FgABCB8* cDNA (which includes an XbaI site) into the 5′ end of the pdr5 terminator using reverse primer (pdrt1R) with primer pdrt2F and repeated with forward primer (pdrt3F; Table S1). The pdr5 promoter (594 bp) and terminator (327 bp) were digested with the restriction enzymes SacI and XbaI/KpnI, respectively. A three-fragment ligation was performed with 50 ng of p416-CYC vector (SacI/XbaI), the N-terminal HA-tagged *FgABCB8* cDNA (126 ng; XbaI; 3770 bp) and the pdr5 promoter fragment (21ng; SacI). The resulting plasmid, p416 pdr5 promoter-N terminal HA-*FgABCB8* cDNA (4864 bp), was digested using XbaI/KpnI and ligated to the pdr5 terminator fragment (XbaI/KpnI; 357bp).

To generate the p416-CYC N-terminal HA-tagged *FgABCB8* cDNA vector the p416-CYC vector was linearized by digesting with XmaI and HindIII (5.4 kb). Amplification of pBluescript SK+(3kb) _cDNAFgABCB8 using primers pdrp1R and GSP3817R (Table S1) was done to insert the HA tag to the N-terminal region, and a XmaI and HindIII restriction site upstream and downstream of the ORF, respectively. The resulting PCR product was digested with XmaI and HindIII and ligated with d p416-CYC XmaI/HindIII vector and N-terminal HA-tagged *FgABCB8* cDNA to generate the yeast expression vector.

The resultant expression vectors p416-pdRr5 promoter-N terminal HA-FgABCB8 cDNA-pdr5 terminator and p416-CYC1 N terminal *FgABCB8* cDNA-CYC terminator were confirmed by sequencing and transformed into yeast AD1234578 (*Δyor1*::*hisG*, *Δsnq2*::*hisG*, *pdr5-Δ2*::*hisG*, *Δpdr10*::*hisG*, *Δpdr11*::*hisG*, *Δycf1*::*hisG*, *Δpdr15*::*hisG*, *pdr1-3*, parental strain US50-18C)(Rogers et al., 2001). The plasmid DNA (1 μg; 5μl) was combined with 3μl of denatured herring testes DNA (10 mg/mL), and 100 μL transformation mix (2M lithium acetate; 50% polyethlyene glycol-3350; and 0.8% β-mercaptoethanol). One large yeast colony grown on yeast peptone dextrose (YPD) agar was suspended into the plasmid: transformation mix and incubated for 30 min on a rotator at 37 °C. The yeast cells were pelleted and re-suspended in 100 μL of sterile MQ water and the total volume was plated onto the selection media, Synthetic Defined agar (SD; 0.2% Yeast Nitrogen Base, 40 mM $(NH_4)^2SO_4$, 2% glucose, 1X Drop out media: all concentrations are in mg/L: 20 L-Adenine hemisulfate, 20 L-Arginine HCL, 20 L-Histidine HCL monohydrate, 30 L-Isoleucine, 100 L-Leucine, 30 L-Lysine HCL, 20 L-

Methionine, 50 L-phenylalanine, 200 L-Threonine, 20 L-Tryptophan, 30 L-Tyrosine, 150 L-Valine) without urea for 72 hrs at 30°C.

Supplemental Figures

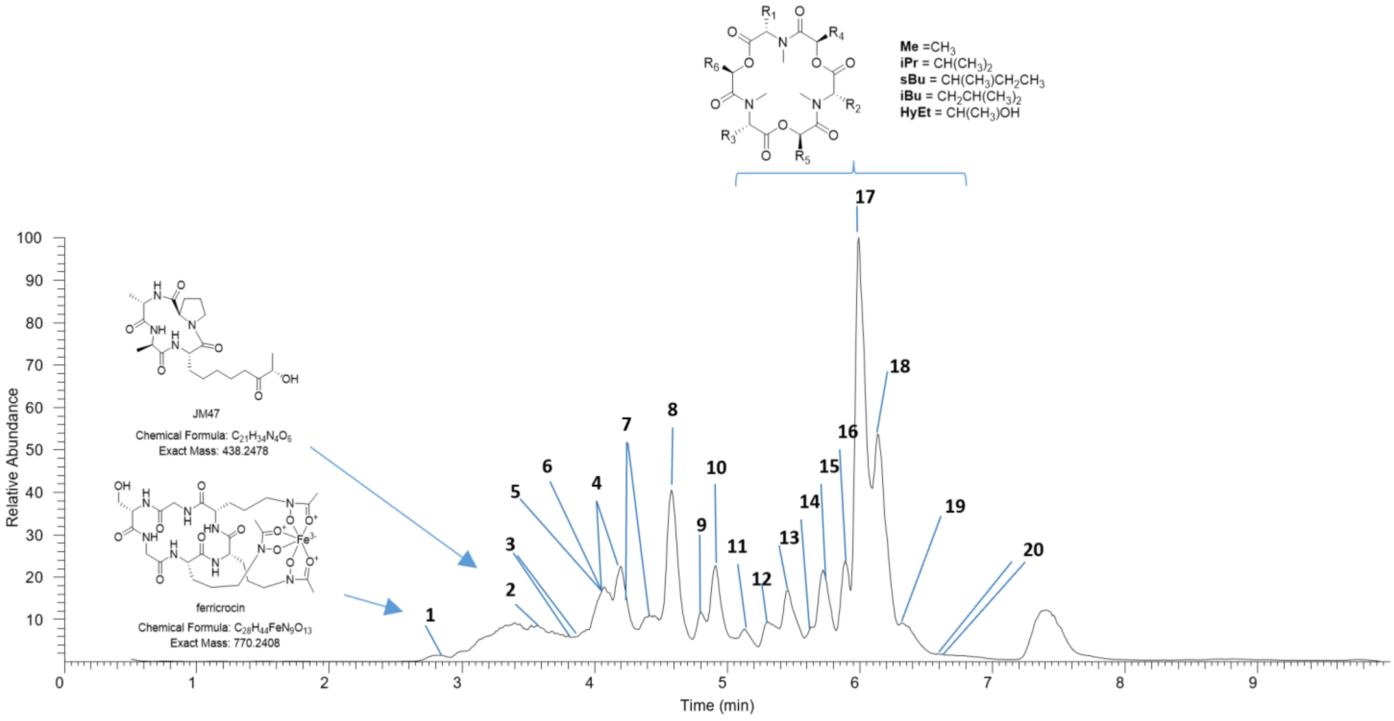

Figure S1. UHPLC-HRMS total ion current (TIC) chromatogram of the mixed methanol extracts of *Fusarium avenaceum* LH27 and LH03. Metabolites associated with numbered peaks are listed in Table S2.

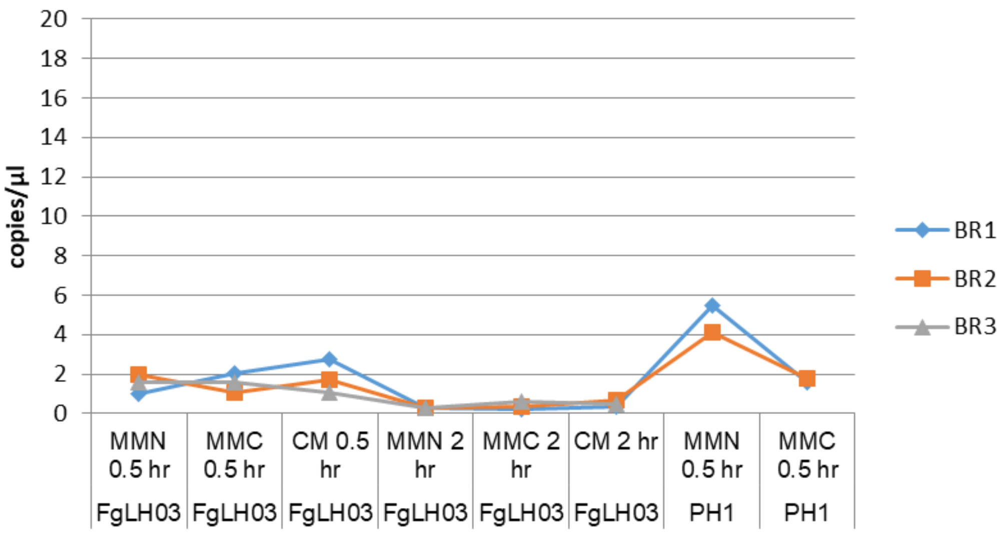

Figure S2. FgABCB8 gene expression in wildtype strains *Fg*LH03 and *Fg*PH1 during growth in complete (CM), carbon limiting (MMC) and nitrogen limiting (MMN) liquid media. After growth in 1st stage media for two days, media was replaced with either CM, MMC, or MMN and mycelia was collected at two time points (30 min and 2 hr). Expression normalized to β-tubulin expressed at 40 copies/µL.

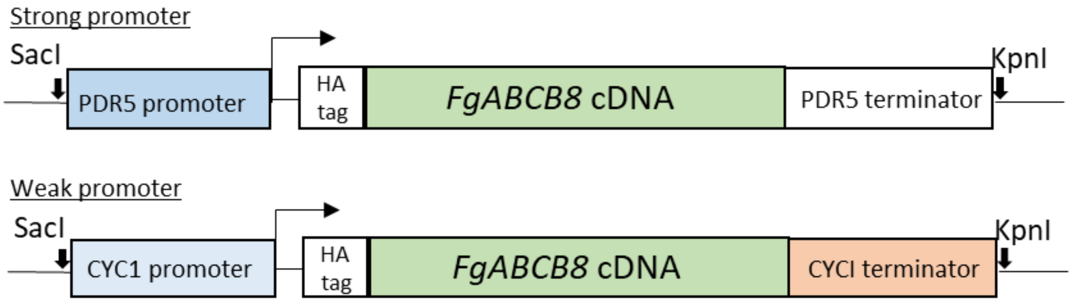

Figure S3. Protein expression vector constructs for use in yeast

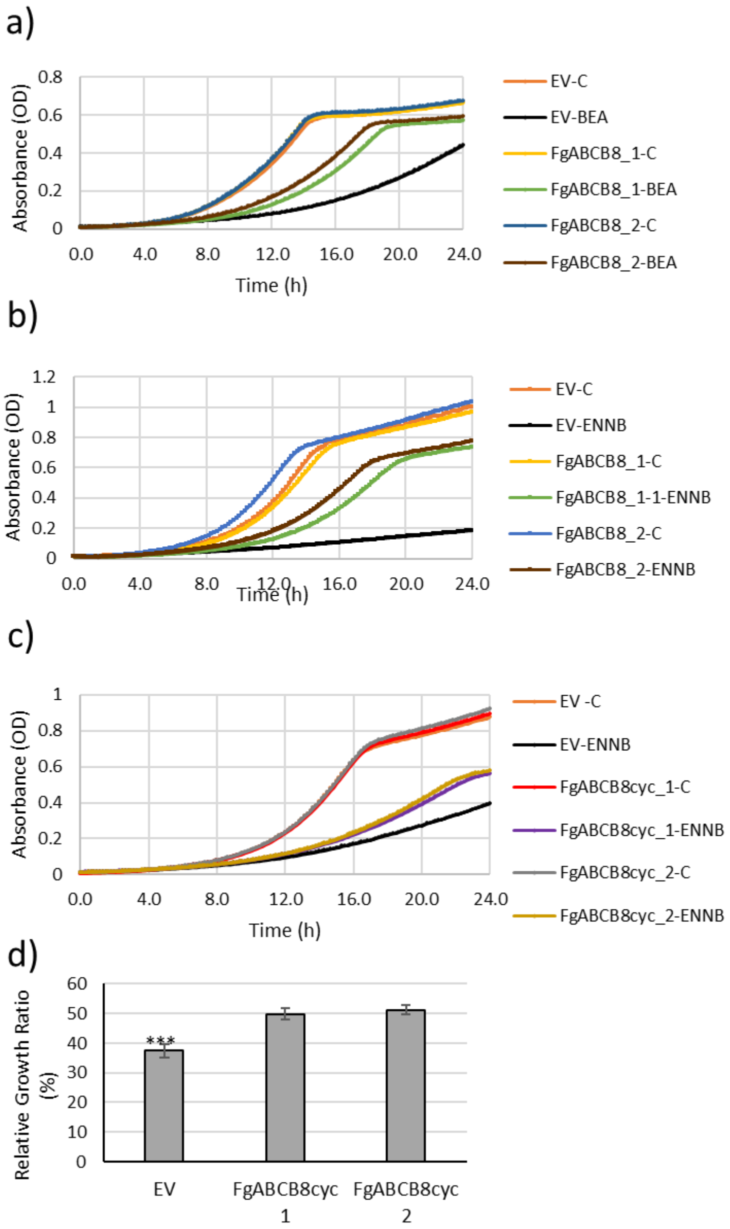

Figure S4. **Expression of FgABCB8 protects yeast from the growth-inhibiting effects of enniatin B1 and beauvericin.** a. AD12345678_EV or AD12345678_*PDR5* promoter-*FgABCB8* (two independent clones) grown in the presence of 10 μM beauvericin (BEA) or control (C); b. AD12345678_EV or AD12345678_*PDR5*promoter-*FgABCB8* (two independent clones) grown in the presence of 10 μM Enniatin B1 (EnnB) or control (C); c. AD12345678_EV or AD12345678_*CYC1*promoter-*FgABCB8* (two independent clones) grown in the presence of 10 μM Enniatin B1 (EnnB) or control (C); d. relative growth ratio of AD12345678_EV and two independent clones of AD12345678_*CYC1*promoter-*FgABCB8* in 10 μM Enniatin B1 relative to control treatment. ***, $p < 0.005$.

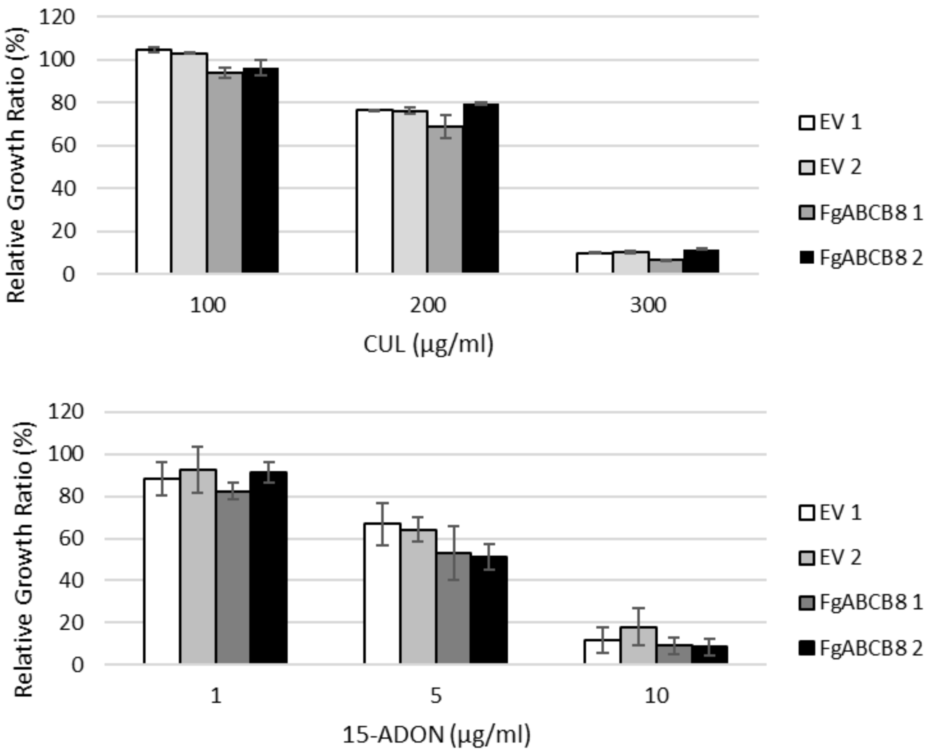

Figure S5. **FgABCB8 expression does not protect yeast against culmorin or 15-acetyl-deoxynivalenol.** AD12345678_EV or AD12345678_*PDR5*promoter-*FgABCB8* (two independent clones) grown in the presence of increasing concentrations of CUL (culmorin) or 15-acetyldeoxynivalenol (15-ADON).

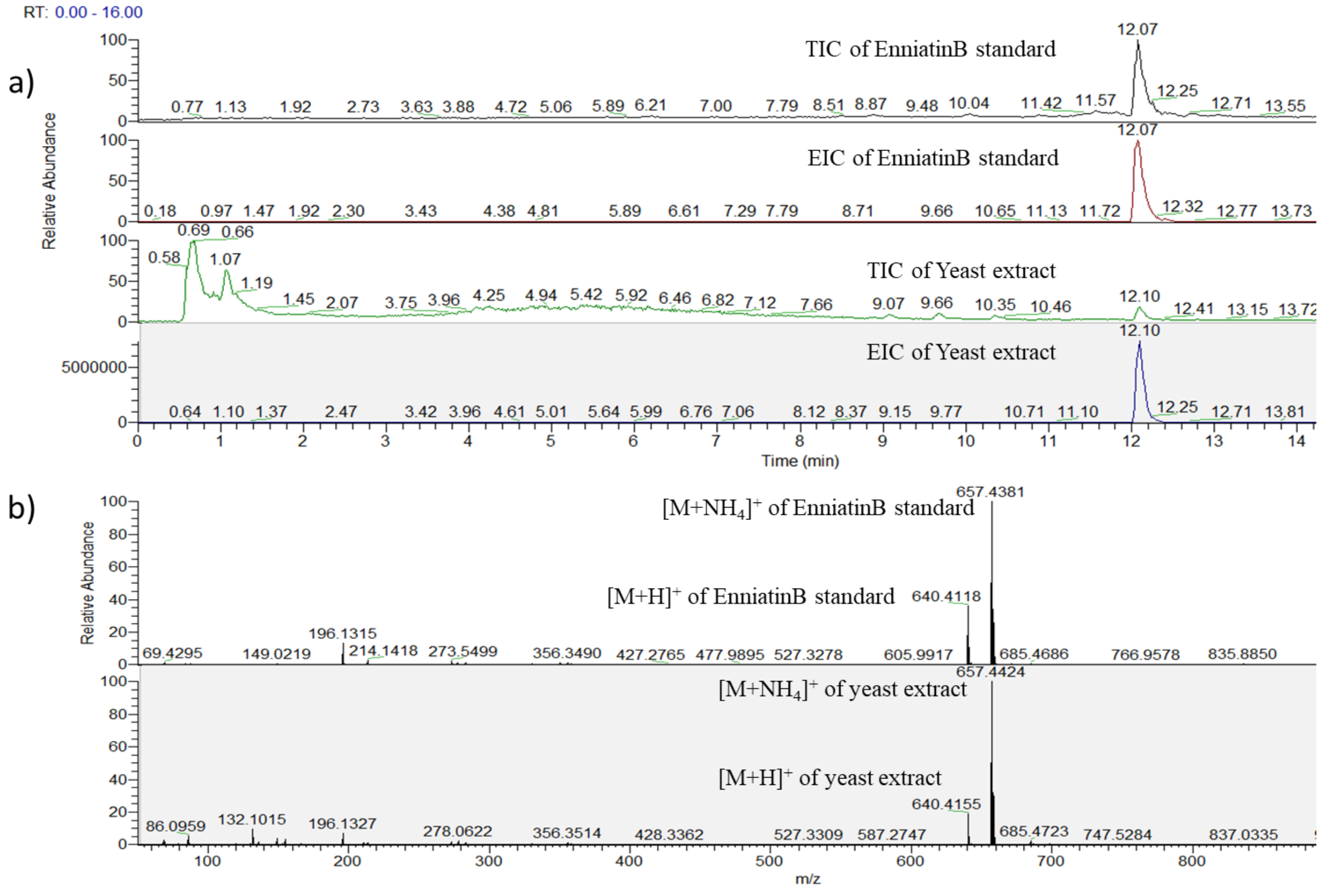

Figure S6. LCMS analysis method of enniatin B. Total ion chromatogram (TIC) and extracted ion chromatogram (EIC) (a) and HR-mass spectra (b) of enniatin B standard and a representative yeast extract from sample YEA_ENNB_4.

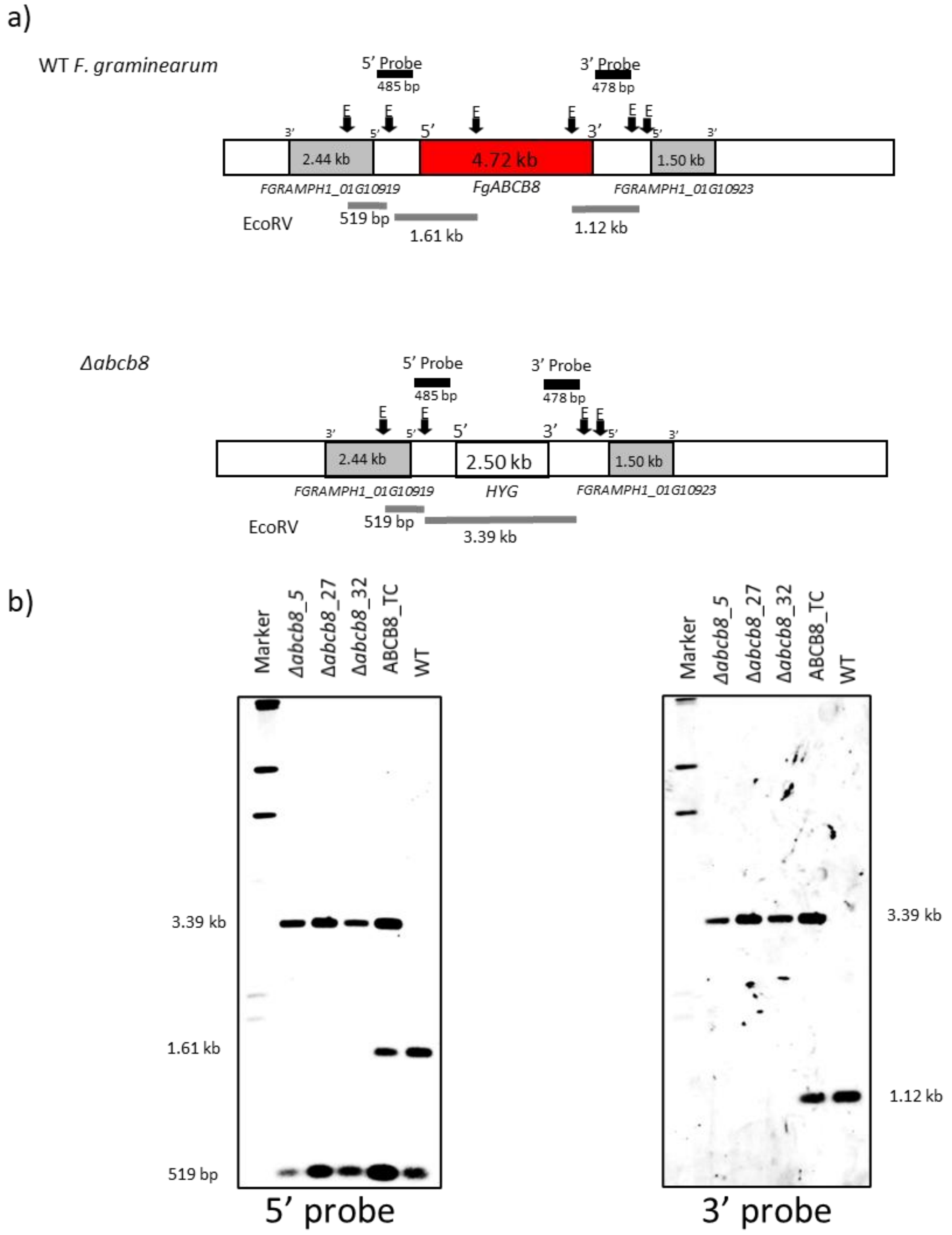

Figure S7. Structure and Southern confirmation of *FgABCB8* gene disruption. a. Genomic structure of wildtype *Fg*LH03 and a *FgABCB8* deletion mutant displaying 5′ and 3′ hybridization probes and predicted EcoRV-restricted band sizes. E, EcoRV restriction sites. b. Southern analysis of genomic DNA of transformant strains and the progenitor wildtype (WT) strain *Fg*LH03 digested with EcoRV and hybridized with the 5′ or 3′ flanking regions shown in panel a.

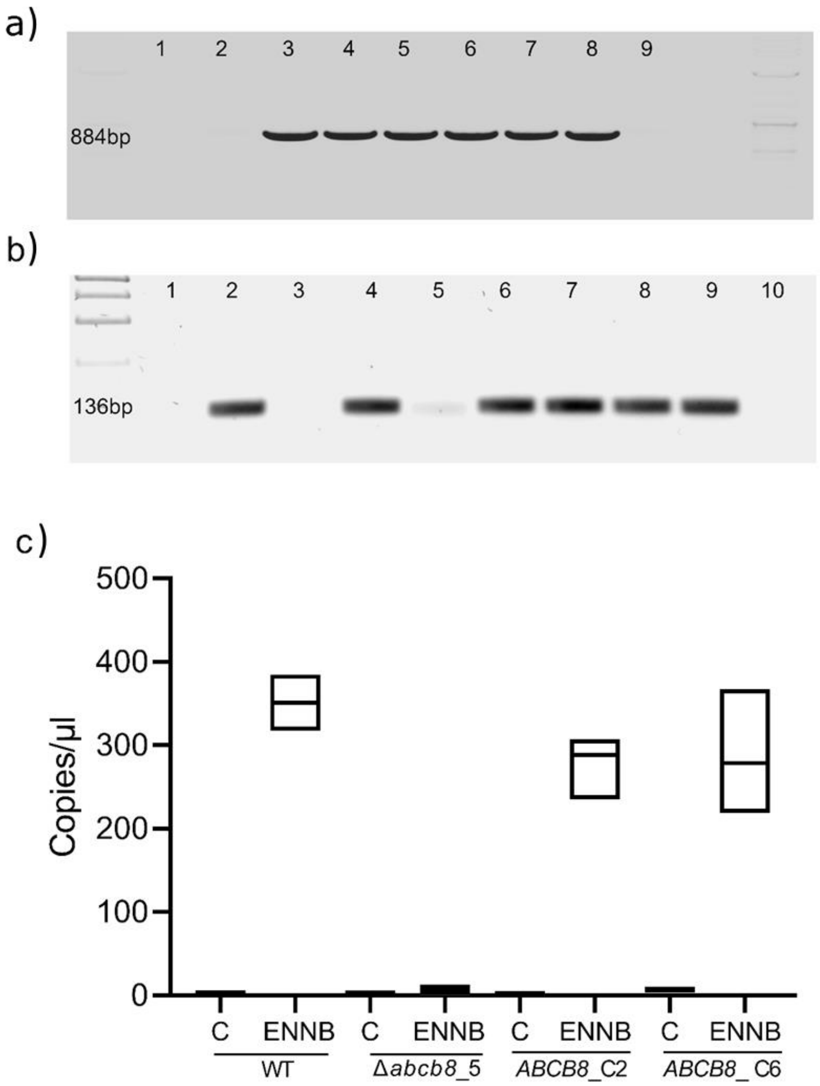

Figure S8. Validation of *FgABCB8* complemented strains. a) Genomic PCR using Fg*ABCB8*-specific primers to confirm gene presence in transgenic strains. Lane 1, Δ*abcb8*_5; lane 2, Δ*abcb8*_32; lane 3, *ABCB8*_TC; lane 4, *ABCB8*_C2; lane 5, *ABCB8*_C4; lane 6, *ABCB8*_C5; lane 7, *ABCB8*_C6; lane 8, WT FgLH03; lane 9, -ve control. b) RT-PCR using RNA extracted from transgenic and wildtype strains after enniatin induction to confirm expression of *FgABCB8*. Lane 1, negative control; lane 2, WT; lane 3, Δ*abcb8*_5; lane 4, *ABCB8*_C2; lane 5, *ABCB8*_C3; lane 6, *ABCB8*_C4; lane 7, *ABCB8*_C5; lane 8, *ABCB8*_C6; lane 9, *ABCB8*_C7; lane 10, no RT control (with WT sample). c) Confirmation of enniatin-inducible expression of *FgABCB8* in two complemented strains by ddPCR. Fungal cultures were spiked with DMSO (C) or 2 µg/mL of enniatin B in DMSO (ENNB) and grown for 2 hours prior to RNA extraction.

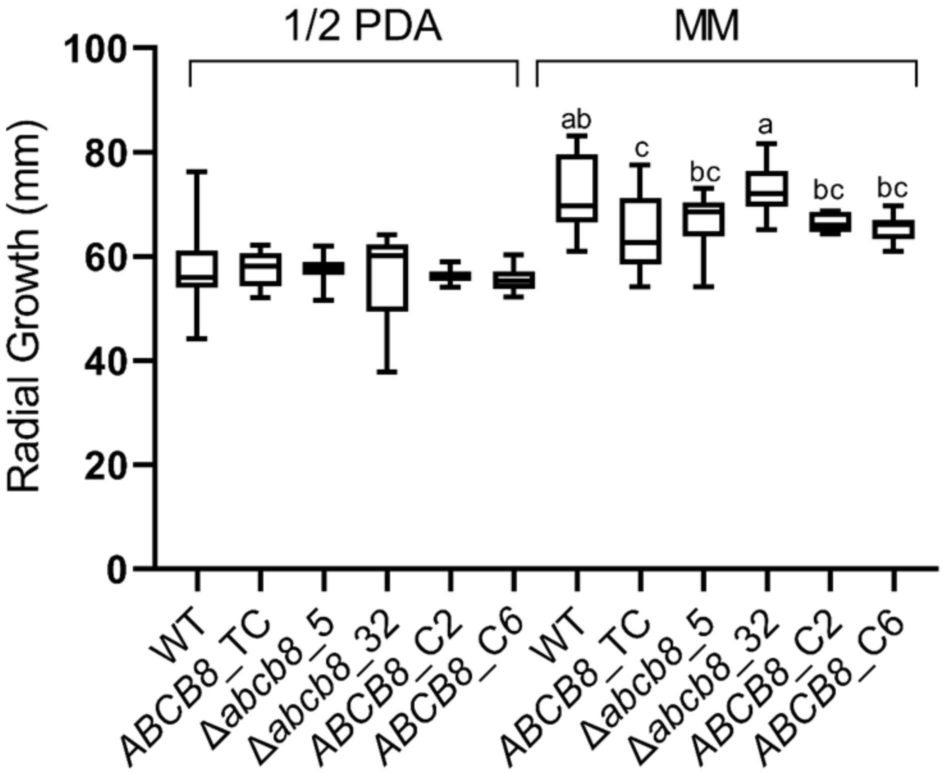

Figure S9: Solid growth assay of wildtype and derived transformants. Radial growth of WT *F. graminearum* (FgLH03), *ABCB8*_TC (transformant control), *ABCB8* deletion mutants *(Δabcb8*) and complemented strains (*ABCB8*_C) on ½ strength potato dextrose agar (1/2 PDA) and minimal media (MM) after four days. No significant differences between strains grown o n 1/2PDA. On MM, different lower case letters indicate significant difference, by Dunnett's multiple comparisons test, $p < 0.05$. Four biological replicates of three technical replicates each.

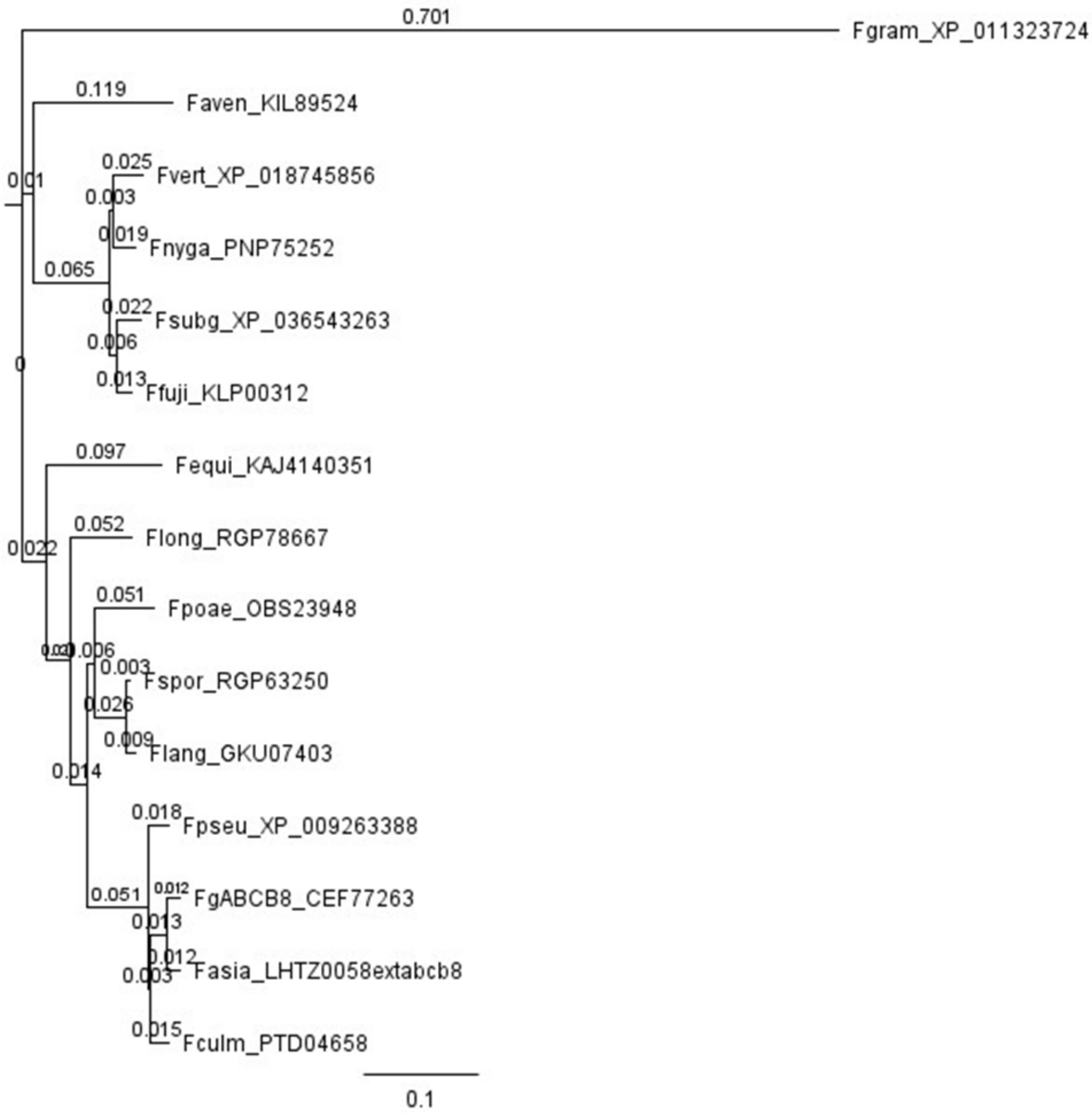

Figure S10: Jukes-Cantor/Neighbor-joining consensus tree of Clustal Omega alignment of FgABCB8 orthologues in representative *Fusarium* cereal pathogens. Branch labels display substitutions per site. Outlier CEF77458 is the closest related *F. graminearum* protein to FgABCB8.
